# Supplementary figures and images for: Human brains construct individualized global rankings from identical few-shot learning input
Source: PLoS Biol. 2026 Apr 13;24(4):e3003756. doi: 10.1371/journal.pbio.3003756 (PMC13108901; doi:10.1371/journal.pbio.3003756)

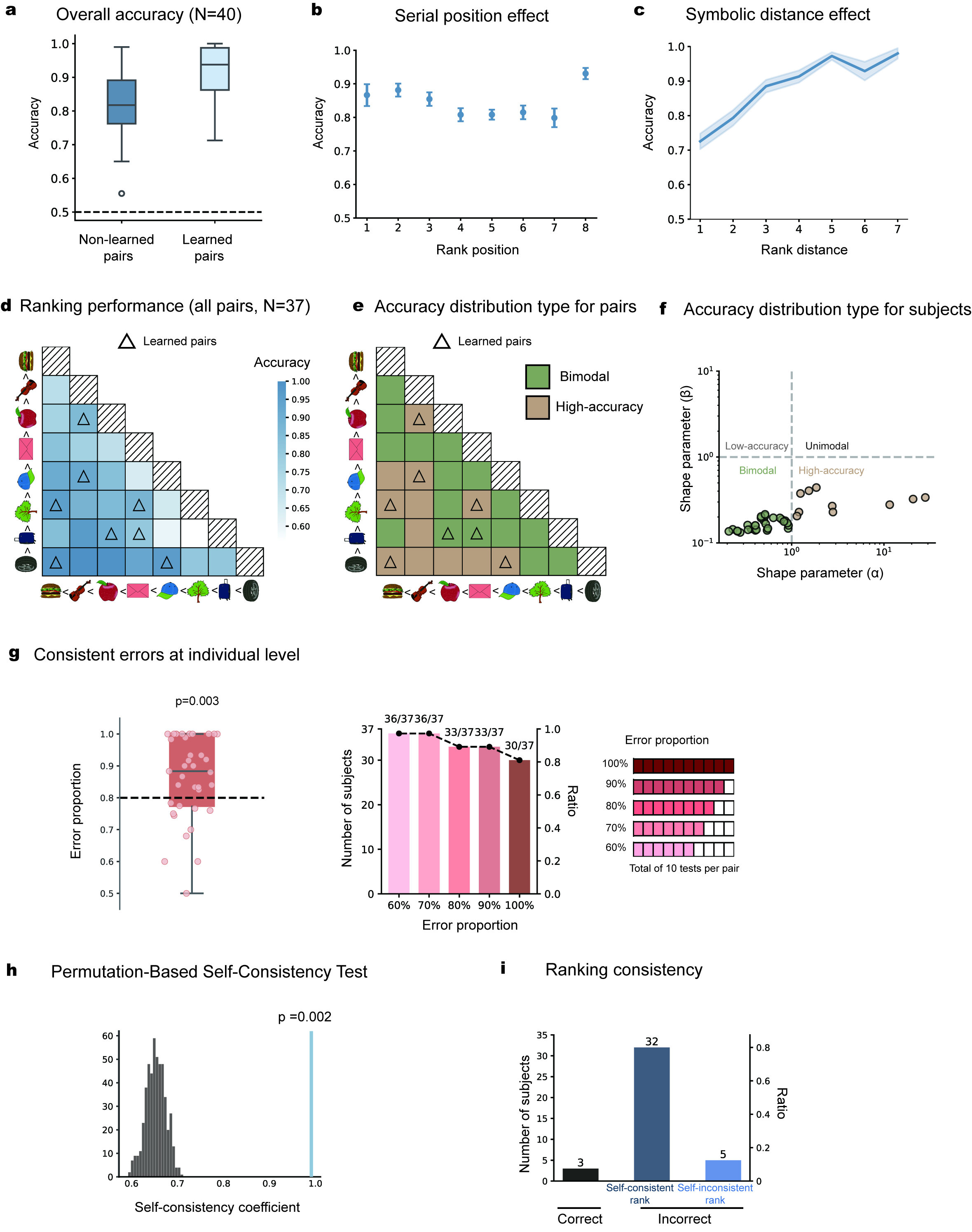

Supplement: S1 Fig — (a) Grand averaged ranking accuracy for learned (light blue; 8 pairs in few-shot learning) and non-learned pairs (dark blue; remaining 20 pairs not directly learned). (b) Grand averaged accuracy as function of rank position (serial position effect). (c) Grand averaged accuracy as function of ranking distance (distance effect). (d) Grand averaged accuracy matrix for all the 24 tested pairs, with row and column denoting corresponding items per pair (Deep-to-light color represents high-to-low accuracy). Eight directly-learned pairs (few-shot learning) were marked by triangles. (e) Beta-distribution model fitting results for each pair (brown: high-accuracy, α > 1, β < 1; green; bimodal distribution, α < 1, β < 1. (f) Beta-distribution model fitting results for each subject. Each point represents the best-fitting α and β parameters for an individual subject. Brown points indicate subjects with bimodal error distributions (α < 1, β < 1); green points indicate subjects exhibiting high-accuracy patterns (α > 1, β < 1). Both α and β are significantly smaller than 1 (one-sample t test, excluding 10 high-accuracy participant as pre-registered; α, t(29) = −12.32, p < 0.001; β, t(29) = −185.69, p < 0.001). (g) Individual-level error consistency (left) and proportion of subjects making consistent error on at least one local pair for different thresholds (0.6 to 1.0). Dots denote individual data. (h) Self-consistency was significantly higher than chance (permutation test: p = 0.002; observed = 0.99). (i) Number of subjects for each self-consistency category. The data underlying this Figure can be found in https://osf.io/gya95/. (TIF) [file pbio.3003756.s001.tif]

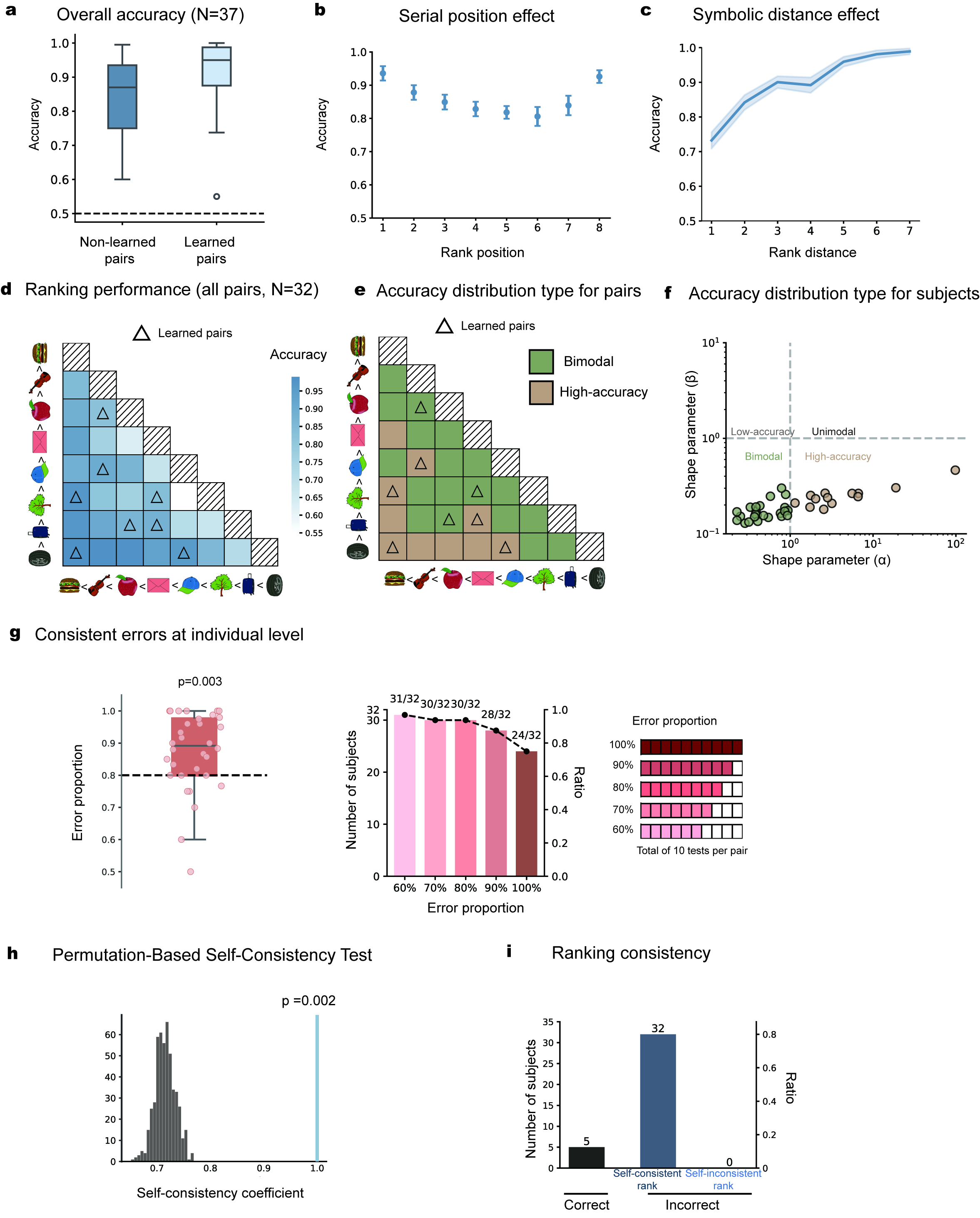

Supplement: S2 Fig — (a) Grand averaged ranking accuracy for learned (dark blue; 8 pairs in few-shot learning) and non-learned pairs (light blue; remaining 20 pairs not directly learned). (b) Grand averaged accuracy as function of rank position (serial position effect). (c) Grand averaged accuracy as function of ranking distance (distance effect). (d) Grand averaged accuracy matrix for all the 24 tested pairs, with row and column denoting corresponding items per pair (Deep-to-light color represents high-to-low accuracy). Eight directly-learned pairs (few-shot learning) were marked by triangles. (e) Beta-distribution model fitting results for each pair (brown: high-accuracy, α > 1, β < 1; green; bimodal distribution, α < 1, β < 1. (f) Beta-distribution model fitting results for each subject. Each point represents the best-fitting α and β parameters for an individual subject. Brown points indicate subjects with bimodal error distributions (α < 1, β < 1); green points indicate subjects exhibiting high-accuracy patterns (α > 1, β < 1). Both α and β are significantly smaller than 1 (one-sample t test, excluding 13 high-accuracy participant as pre-registered; α, t(23) = −9.27, p < 0.001; β, t(23) = −97.30, p < 0.001). (g) Individual-level error consistency (left) and proportion of subjects making consistent error on at least one local pair for different thresholds (0.6 to 1.0). Dots denote individual data. (h) Self-consistency was significantly higher than chance (permutation test: p = 0.002; observed = 1.00). (i) Number of subjects for each self-consistency category. The data underlying this Figure can be found in https://osf.io/gya95/. (TIF) [file pbio.3003756.s002.tif]

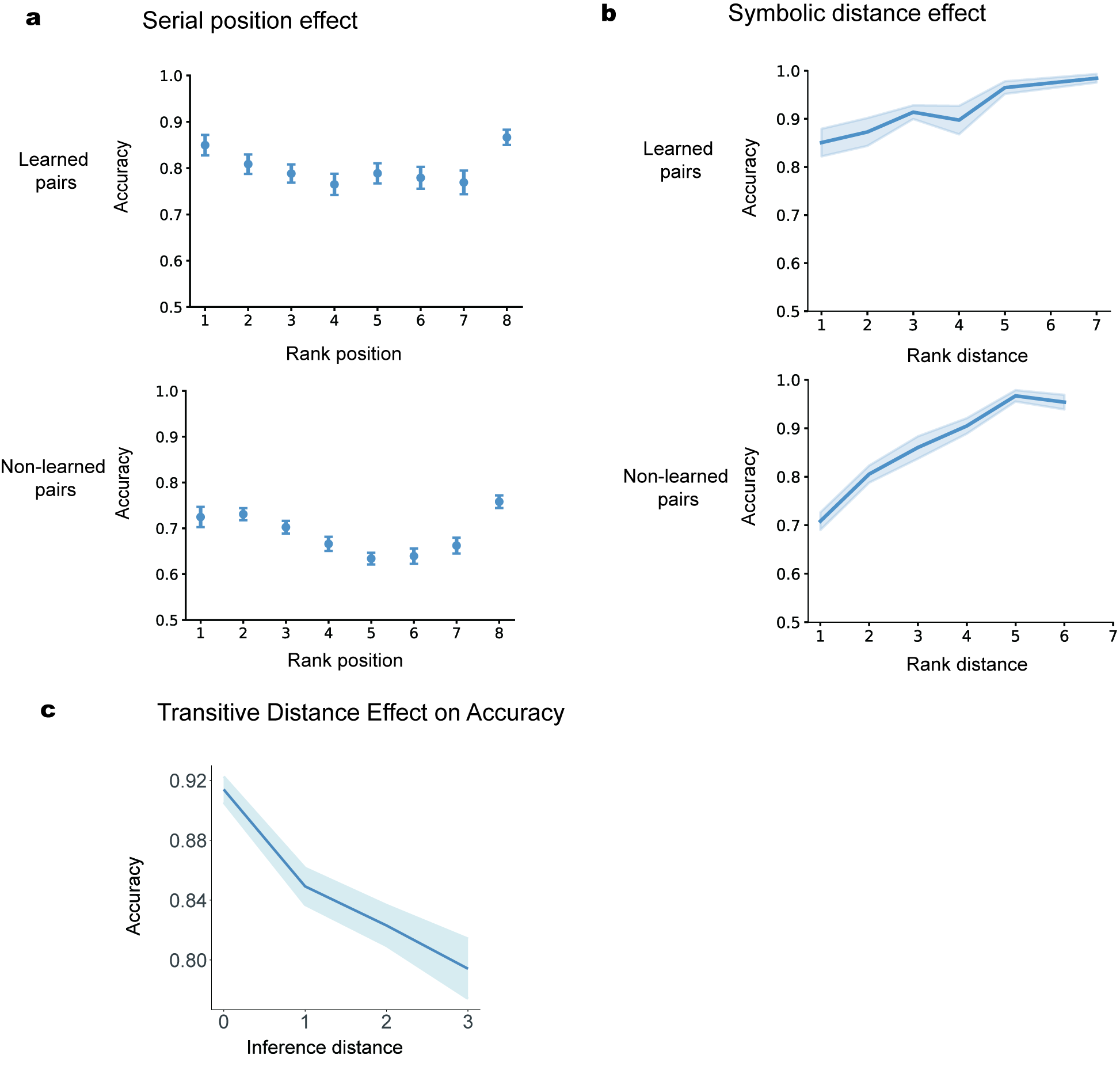

Supplement: S3 Fig — (a) Serial position effect. Upper panel (learned): F(7, 532) = 11.87, p < 0.001; lower panel (non-learned): F(7, 532) = 11.87, p < 0.001. (b) Symbolic distance effect. Upper panel (learned): slope coefficient = 0.02, t(76) = 4.556, p < 0.001; lower panel (non-learned): slope coefficient = 0.05, t(76) = 14.141, p < 0.001. (c) Transitive distance effect is significant without controlling for rank distance linear mixed-effect model; F(1, 1463.00) = 6.37, p = 0.012) but not significant after control (F(1, 1463.00) = 0.17, p = 0.68). Pairs with 0 transitive distance is excluded from this analysis as they are directly learned pairs. The data underlying this Figure can be found in https://osf.io/gya95/. (TIF) [file pbio.3003756.s003.tif]

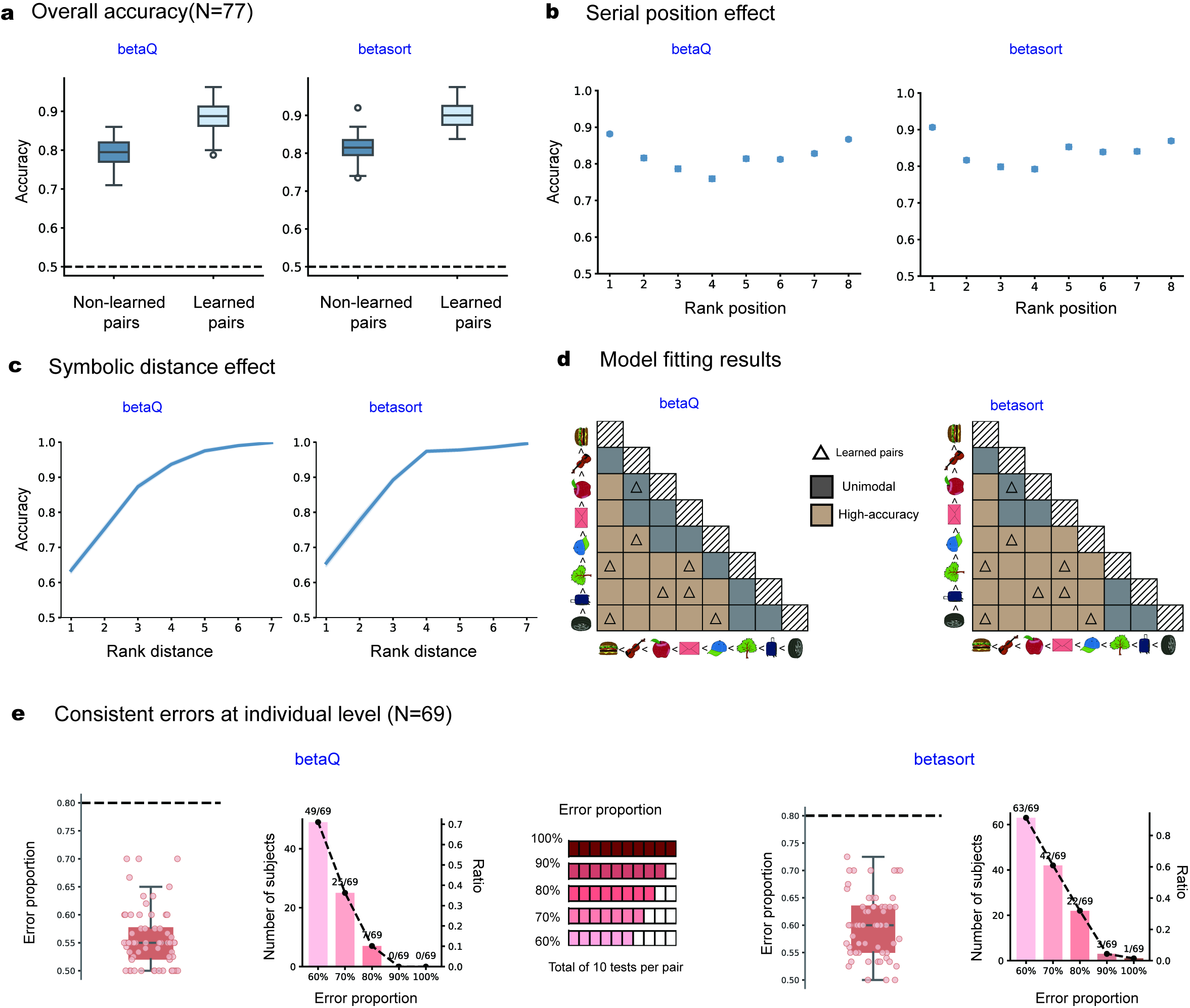

Supplement: S4 Fig — (a) Grand averaged ranking accuracy for learned (dark blue; 8 pairs in few-shot learning) and non-learned pairs (light blue; remaining 20 pairs not directly learned). (b) Grand averaged accuracy as function of rank position (serial position effect). (c) Grand averaged accuracy as function of ranking distance (distance effect). (d) Beta-distribution fitting results for each pair (brown: high-accuracy, α > 1, β < 1; gray; unimodal distribution, α > 1, β > 1). (e) Individual-level error consistency based on betaQ (Left) and betasort (Right) models. Left: grand averaged error consistency (>0.8, one-sample t test, p > 0.05). dots denote individual data. Right: number and proportion of subjects making consistent error on at least one local pair for different thresholds (0.6 to 1.0). The data underlying this Figure can be found in https://osf.io/gya95/. (TIF) [file pbio.3003756.s004.tif]

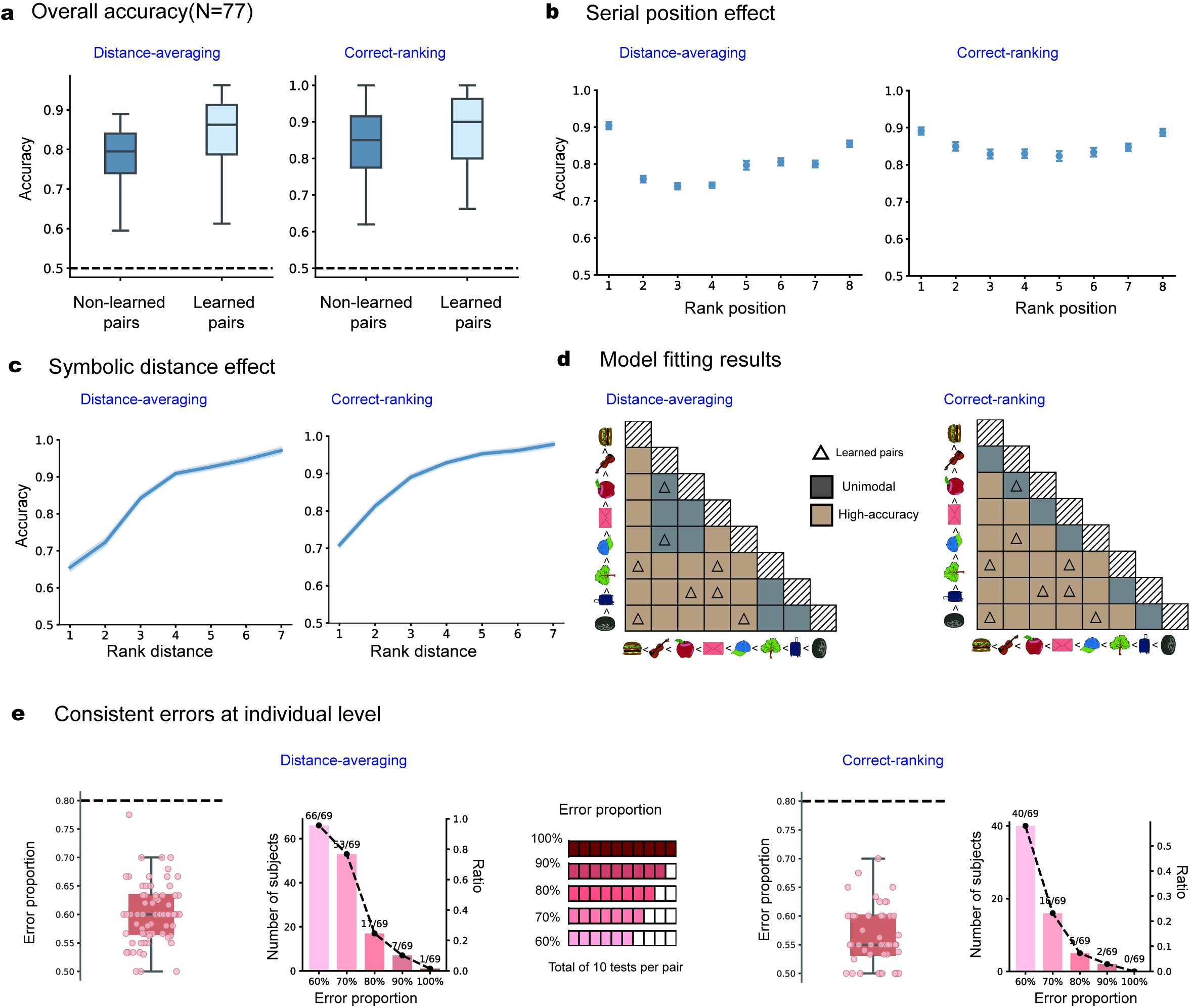

Supplement: S5 Fig — (a) Grand averaged ranking accuracy for learned (dark blue; 8 pairs in few-shot learning) and non-learned pairs (light blue; remaining 20 pairs not directly learned). (b) Grand averaged accuracy as function of rank position (serial position effect). (c) Grand averaged accuracy as function of ranking distance (distance effect). (d) Beta-distribution fitting results for each pair (brown: high-accuracy, α > 1, β < 1; gray; unimodal distribution, α > 1, β > 1). (e) Individual-level error consistency based on Distance-averaging (Left) and correct-ranking (Right) models. Left: grand averaged error consistency (>0.8, one-sample t test, p > 0.05). dots denote individual data. Right: number and proportion of subjects making consistent error on at least one local pair for different thresholds (0.6 to 1.0). The data underlying this Figure can be found in https://osf.io/gya95/. (TIF) [file pbio.3003756.s005.tif]

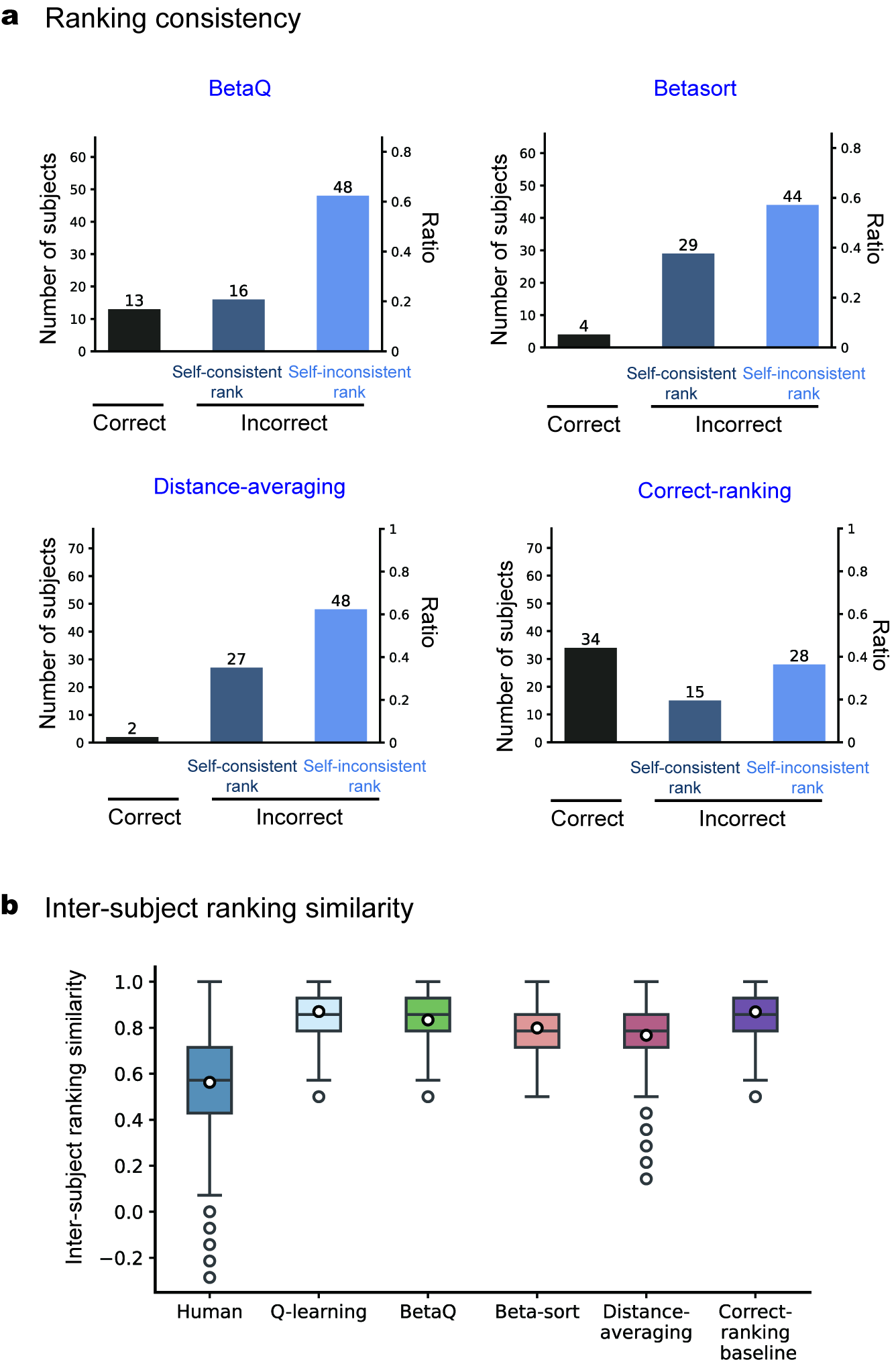

Supplement: S6 Fig — (a) Number of subjects by self-Consistency category for BetaQ, Betasort, Distance-Averaging, and Correct-Ranking Models. (b) Inter-subject ranking similarity for Human, Q-learning, BetaQ, Betasort, Distance-Averaging, and Correct-Ranking Models. The data underlying this Figure can be found in https://osf.io/gya95/. (TIF) [file pbio.3003756.s006.tif]

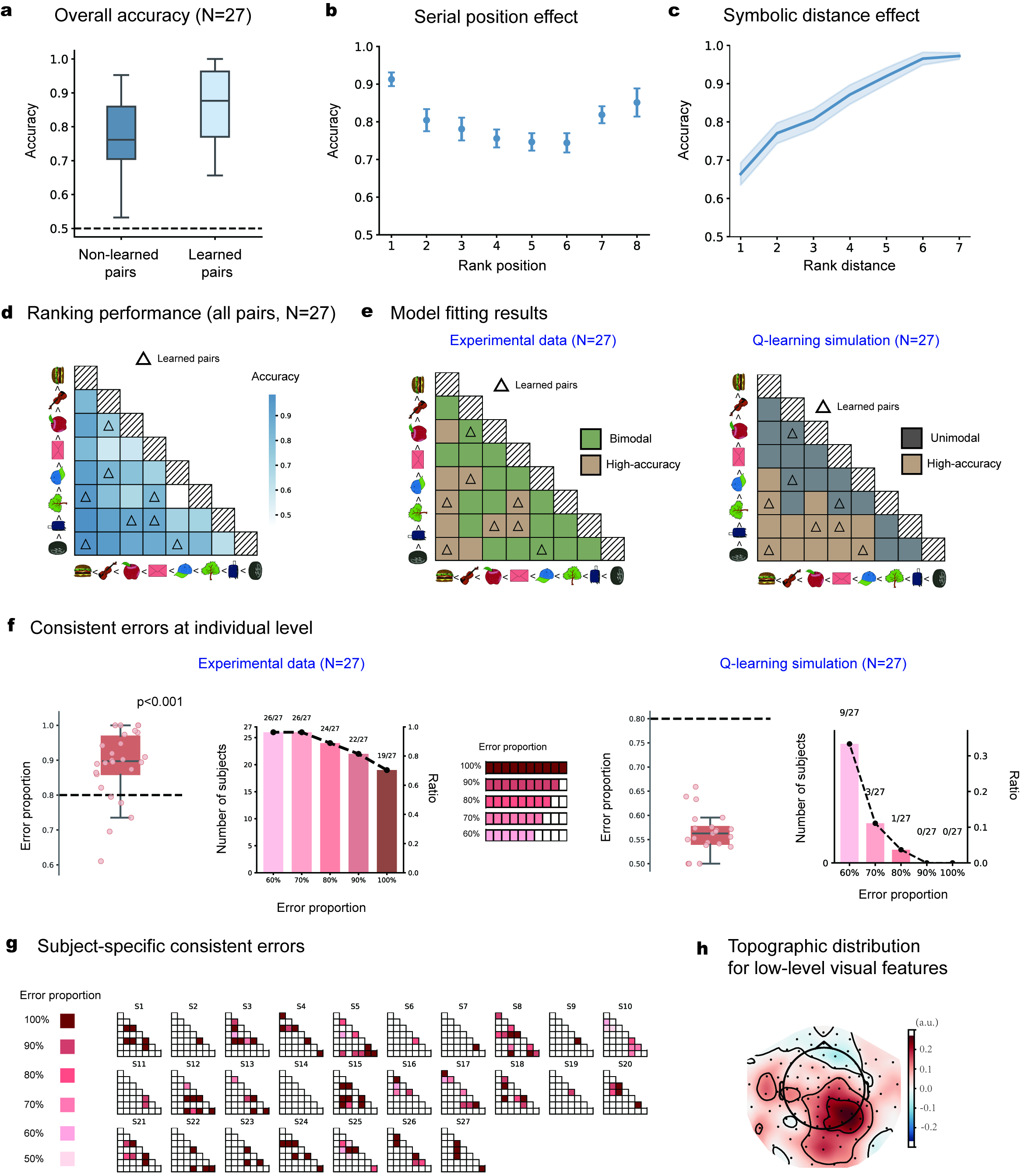

Supplement: S7 Fig — (a) Grand averaged ranking accuracy for learned (dark blue; 8 pairs in few-shot learning) and non-learned pairs (light blue; remaining 20 pairs not directly learned). (b) Grand averaged accuracy as function of rank position (serial position effect). (c) Grand averaged accuracy as function of ranking distance (distance effect). (d) Grand averaged accuracy matrix for all the 28 tested pairs, with row and column denoting corresponding items per pair (Deep-to-light color represents high-to-low accuracy). Eight directly-learned pairs (few-shot learning) were marked by triangles. (e) Beta-distribution model fitting results for each pair (brown: high-accuracy, α > 1, β < 1; green; bimodal distribution, α < 1, β < 1; gray; unimodal distribution, α > 1, β > 1) for experimental data (left) and Q-learning model (right). Note that simulation results support unimodal distribution that diverges from bimodal experimental findings. (f) Individual-level error consistency and proportion of subjects making consistent error on at least one local pair for different thresholds (0.6 to 1.0), for experimental data (left) and Q-learning model (right). Dots denote individual data. (g) Consistent local errors for each subject. (h) Topographic distributions for low-level visual features using sensor-level search light analysis. The data underlying this Figure can be found in https://osf.io/gya95/. (TIF) [file pbio.3003756.s007.tif]

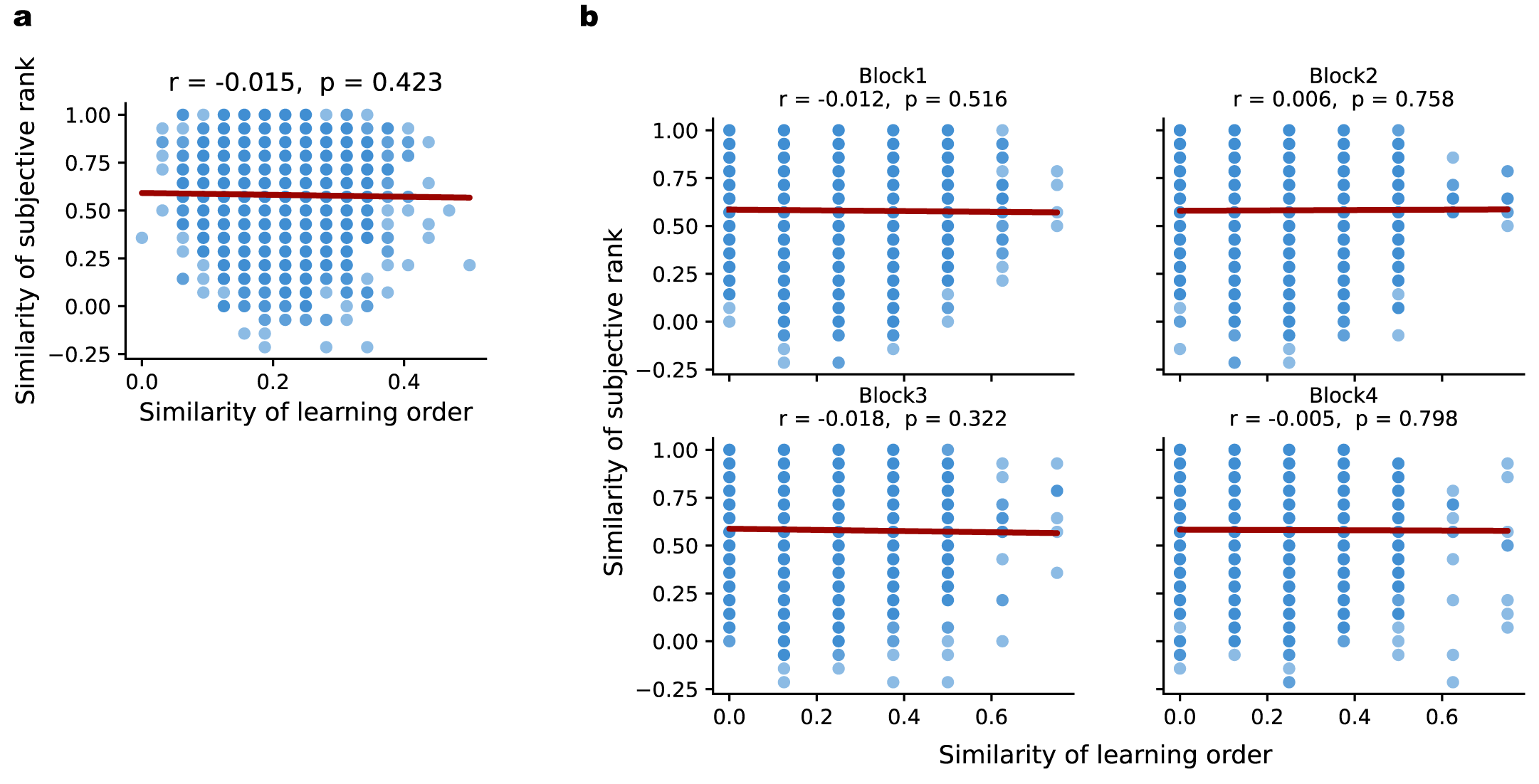

Supplement: S8 Fig — (a) Overall correlation across all learning blocks revealed no significant association (r = −0.015, p = 0.423). (b) Separate analyses for each learning block confirmed the null relationship (Block 1: r = −0.012, p = 0.516; Block 2: r = 0.006, p = 0.758; Block 3: r = −0.018, p = 0.322; Block 4: r = −0.005, p = 0.798). The data underlying this Figure can be found in https://osf.io/gya95/. (TIF) [file pbio.3003756.s008.tif]

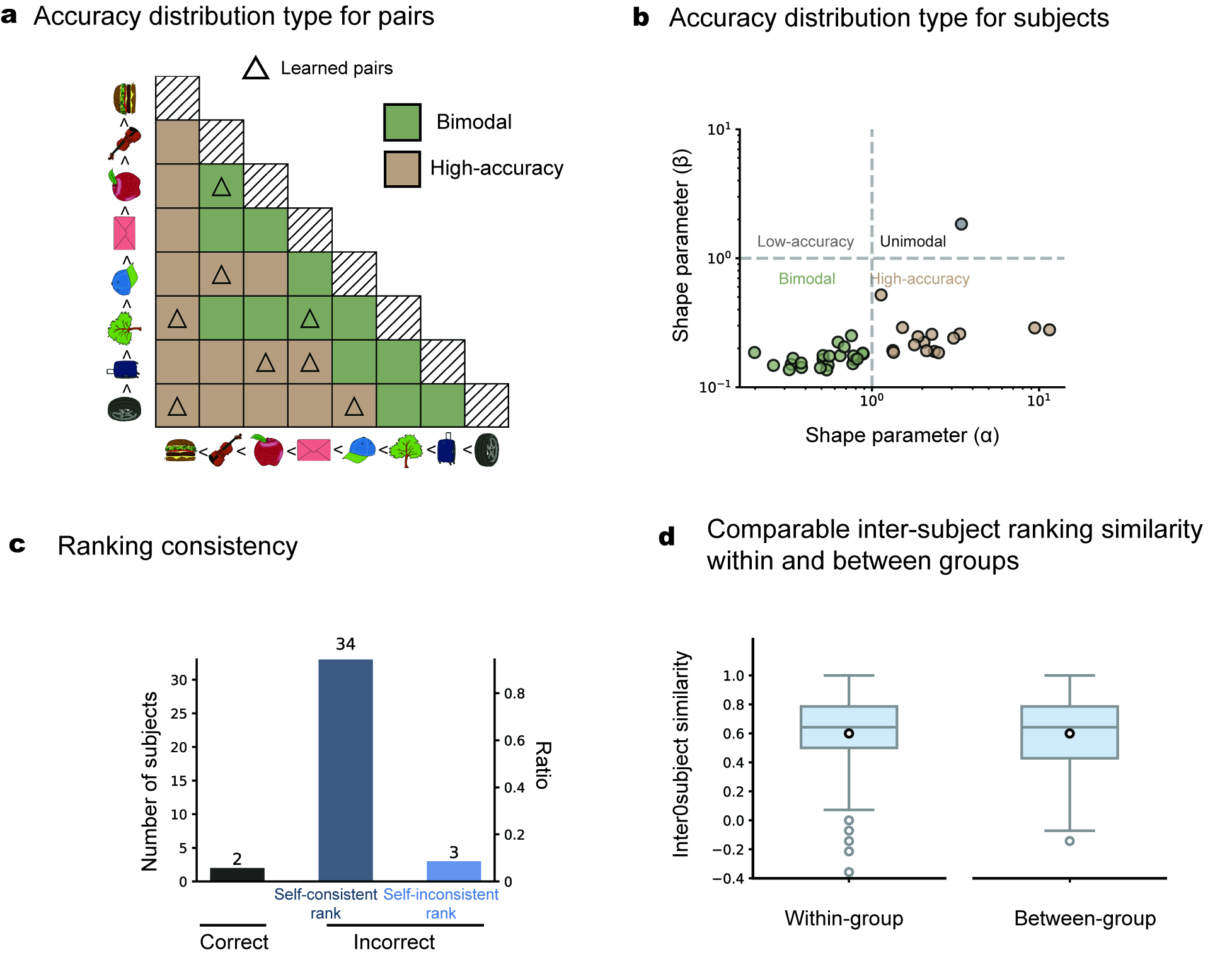

Supplement: S9 Fig — (a) Beta-distribution model fitting results for each pair (brown: high-accuracy, α > 1, β < 1; green; bimodal distribution, α < 1, β < 1). (b) Beta-distribution model fitting results for each subject. Each point represents the best-fitting α and β parameters for an individual subject. Brown points indicate subjects with bimodal error distributions (α < 1, β < 1); green points indicate subjects exhibiting high-accuracy patterns (α > 1, β < 1). Both α and β are significantly smaller than 1 (one-sample t test, excluding 15 high-accuracy participant as pre-registered; α, t(23) = −10.11, p < 0.001; β, t(23) = −142.26, p < 0.001). (c) Number of subjects for each self-consistency category. (d) Independent samples t test revealed comparable ranking consistency among participants who learned the same stimulus set (within-group) versus different stimulus sets (between-group), t = 0.02, p = 0.99. This null result suggests that the specific stimulus set used during learning did not systematically influence participants’ subsequent ranking strategies. The data underlying this Figure can be found in https://osf.io/gya95/. (TIF) [file pbio.3003756.s009.tif]

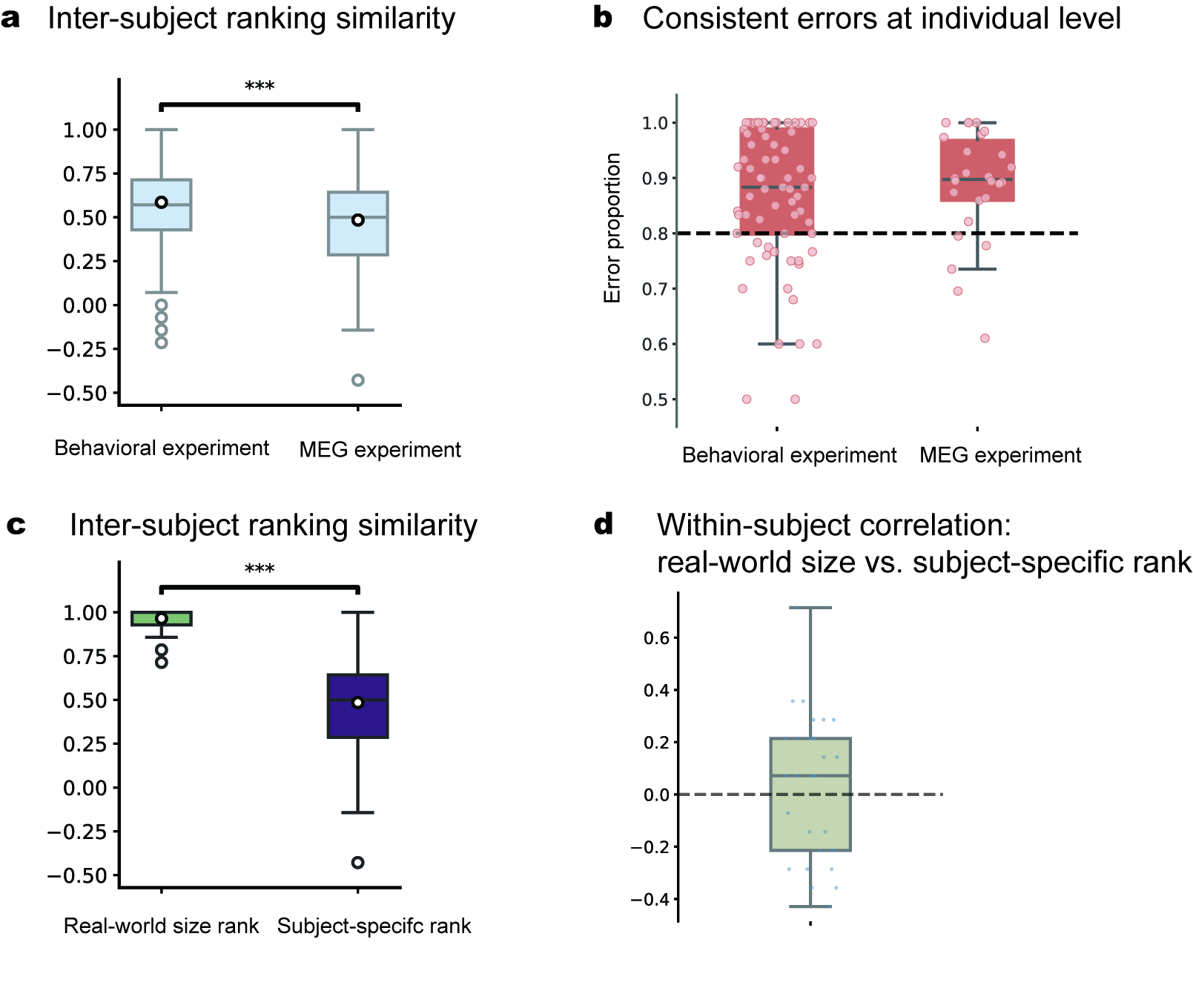

Supplement: S10 Fig — (a) Lower inter-subject ranking similarity in MEG post-learning phase compared to behavioral experiment. (b) Consistent errors at individual level in MEG post-learning phase compared to behavioral experiment. (c) Lower inter-subject ranking similarity in MEG post-learning phase compared to MEG pre-learning phase. (d) Within-subject correlations between real-world size rankings and individually reconstructed subjective rankings were essentially zero (mean r = 0.03, 95% CI [−0.06, 0.13]). The data underlying this Figure can be found in https://osf.io/gya95/. (TIF) [file pbio.3003756.s010.tif]

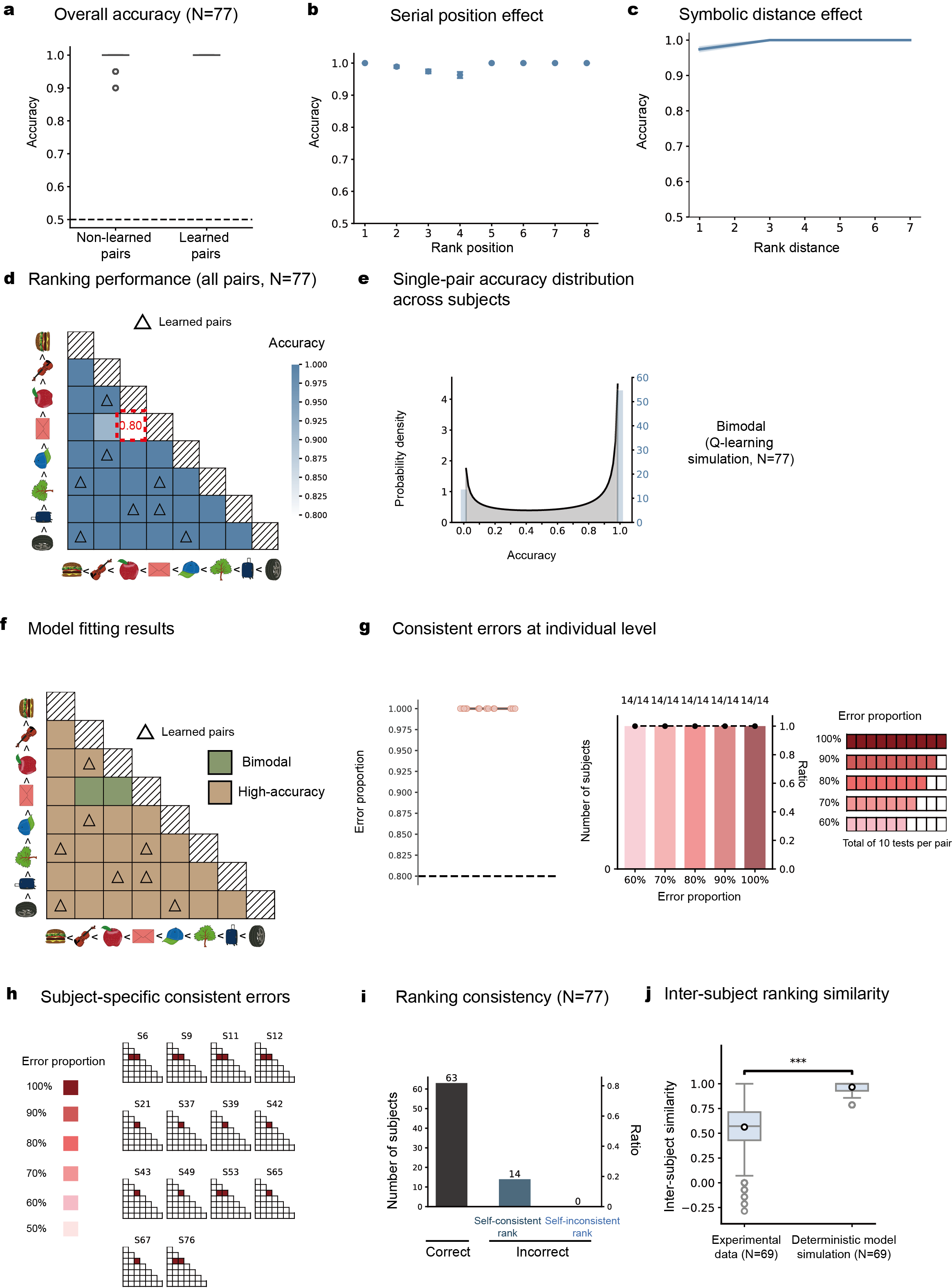

Supplement: S11 Fig — (a) Grand averaged ranking accuracy for learned (dark blue; 8 pairs in few-shot learning) and non-learned pairs (light blue; remaining 20 pairs not directly learned). (b) Grand averaged accuracy as function of rank position (serial position effect). (c) Grand averaged accuracy as function of ranking distance (distance effect). (d) Grand averaged accuracy matrix for all the 28 tested pairs, with row and column denoting corresponding items per pair (deep-to-light color represents high-to-low accuracy). Eight directly-learned pairs (few-shot learning) were marked by triangles. Red dotted box highlights the exemplar pair in e. (e) Observed bimodal distribution, i.e., some subjects consistently made correct inference (3rd < 4th), while others made robust errors (3rd > 4th). (f) Beta-distribution fitting results for each pair (brown: high-accuracy, α > 1, β < 1; gray; unimodal distribution, α > 1, β > 1). (g) Individual-level error consistency based on Distance-averaging (Left) and correct-ranking (Right) models. Left: grand averaged error consistency (>0.8, one-sample t test, p < 0.05). dots denote individual data. Right: number and proportion of subjects making consistent error on at least one local pair for different thresholds (0.6 to 1.0). (h) Local error pattern for each subject. Blank tiles in the lower triangular matrices denote correct pairs (accuracy > 50%). Red tiles denote error pairs (deep-to-light color represents high-to-low error proportion). (i) Number of subjects for each self-consistency category. (j) Inter-subject ranking similarity for experimental data and Deterministic simulation (two-sample t test, p < 0.001). The data underlying this Figure can be found in https://osf.io/gya95/. (TIF) [file pbio.3003756.s011.tif]
