# Supplementary material for: Human brains construct individualized global rankings from identical few-shot learning input
Source: PLoS Biol. 2026 Apr 13;24(4):e3003756. doi: 10.1371/journal.pbio.3003756 (PMC13108901; doi:10.1371/journal.pbio.3003756)
Supplement: Attachment — Submitted filename: LiuWangLuo.2025.Response to reviews.docx [file pbio.3003756.s014.docx]

Responses to reviewers

## Reviewer #1

This study provides potentially valuable insights into the constructive and generative nature of human relational learning under sparse data conditions. The use of a preregistered few-shot learning paradigm combined with behavioral experiments and MEG recordings is a novel methodological approach, allowing for both behavioral and neural evidence to support the findings. The study challenges existing models of relational learning by demonstrating that individuals construct idiosyncratic global rankings even when exposed to identical learning inputs, highlighting the role of individual inductive biases and memory constraints.

Thank you for your kind appreciation of this work and for the clear and accurate summary of our findings.

My general concern is that, throughout the paper, the rationale for the experimental design, the description of results, and the interpretation of those results need to be described in much greater detail with much more attention to the logical flow connecting the design, results and conclusions. I have two specific sets of concerns:

First, the authors have invented a novel way to train ordinal relations, but it isn't clear why they did this or what they expected to find. The conventional approach is to train adjacent pairs. Given a list of n items, if all n-1 adjacent pairs are trained, then the participant has complete information about the list order. However, in the current study, the authors train non-adjacent pairs but also provide a cue signaling the distance between the items in each pair. The authors do not fully explain the rationale for their method, but they indicate that they believe that participants (P's) received only partial information about the list order, leading them to make persistent idiosyncratic errors. I do not believe this is a correct description of the task or the P's behavior. I believe that, by providing 8 non-adjacent pairs plus relative distance, the authors' method presents information that uniquely specifies the order of the 8 list items. Hence, their method provides the same information as the conventional adjacent-pair method, just in a less direct manner. I do not believe that the authors can claim that their method presents "partial pairs" if it in fact presents the same information as a complete set of adjacent pairs. Any idiosyncrasy in behavioral responses could be due to P's inability to extract the information available rather than their filling in missing information.

**Reply:** We thank the reviewer for this insightful and fundamental critique, which has substantially improved the conceptual clarity and theoretical framing of our study. We agree that our original terminology was imprecise and potentially misleading.

As the reviewer correctly noted, the eight pairs presented during learning, together with their distance information, in principle contain sufficient information to uniquely determine the complete ranking, as in the conventional adjacent-pair method. We fully agree with this point. To avoid confusion, in the revised manuscript we have replaced the term “*partial pair*” with "*observed pairs*" (referring to the eight pairs directly presented during learning) and named the remaining pairs as "*inferred pairs*" (the 20 pairs that must be inferred during testing).

Building on this point, the reviewer further suggested that any idiosyncrasy in behavioral response is largely due to participants’ inability to extract available information rather than filling in missing information. We agree with this view to some extent. However, we would add that, in practice, although the presented information uniquely determines the global ranking in principle, extracting or inferring the underlying global structure still requires complex integration and inference processes that may exceed participants' cognitive resources, particularly under few-shot conditions (see possible interpretations below). In other words, echoing the reviewer’s point, the key issue is not just about missing information per se, but about how the brain responds when information integration exceeds cognitive capacity. In this sense, we suggest that the observed idiosyncrasy rankings arise from a combination of limitations in information extraction, integration, and inference.

Specifically, we propose two contrasting accounts of relational inference under cognitively demanding few-shot conditions: *independent-value account* and *constructive ranking account*. Under the first account, which underlies most computational models of transitive inference, each item is represented and updated independently as a point on a latent value axis, with no constraints imposed by a coherent global ranking structure. In contrast, the latter account proposes that individuals actively construct a unified global ranking, which serves as an inductive bias to constrain learning of local pairs. Our empirical results support the latter account: participants exhibit stable, self-consistent, yet idiosyncratic subjective ranking, even from identical few-shot learning.

In response, we have substantially revised the *Introduction* (L67-93) to more explicitly ground our hypotheses in the framework of few-shot learning, emphasizing the inherent cognitive resource limitations and the role of inductive biases in relational inference under limited exposure conditions. We believe this revision has led to a clearer articulation of the study’s rationale and a more precise interpretation of our findings. We are grateful to the reviewer for raising this concern, which we believe has fundamentally strengthened the manuscript.

Introduction L69-95

Because ranking involves stochastic relational inference under uncertainty (Lake et al., 2015; Tenenbaum et al., 2011), we hypothesized that inductive biases are engaged during few-shot learning of local pairwise relations. Under sparse evidence and limited computational resources, relational inference is therefore not a passive accumulation of evidence but is shaped by the active construction of a global ranking structure within each individual—an account we refer to as the *constructive ranking account* (Fig 1a, right). According to this account, individuals actively construct a unified and idiosyncratic global ranking from few-shot experience, which serves as an inductive bias to constrain learning of local pairs. As a result, even when exposed to identical local pairwise information, individuals tend to form stable and self-consistent, yet idiosyncratic, global rankings that deviate from the ground truth. This account contrasts sharply with the classical *independent-value account* (Fig 1a, left), previously used to explain transitive inference (Ciranka et al., 2022; Jensen et al., 2015). Under this account, each item is represented and updated independently as a point on a latent value axis, with no constraints imposed by a global ranking structure.

A classical framework for studying relational learning is transitive inference (Acuna & Sanes, 2002; Bryant & Trabasso, 1971; Piaget, 1923; Vasconcelos, 2008), in which relationships between non-adjacent items (e.g., A < C) are inferred from relationships between adjacent items (e.g., A < B, B < C). A related phenomenon, list linking (Nelli et al., 2023; Treichler & Van Tilburg, 1996; Wei et al., 2014), occurs when two independently learned sequences are rapidly integrated into a single ordered list after learning a single bridging relation. Numerous computational and neural network models have been proposed to account for such relational learning (Ciranka et al., 2022; Delius & Siemann, 1998; Jensen et al., 2015; Mannella & Pezzulo, 2024; Wynne, 1995; Kay et al., 2024; Miconi & Kay, 2025; Mitchell, 2007; Nelli et al., 2023). Here, we adapted the transitive inference paradigm within a few-shot learning framework to directly examine the role of inductive bias in relational inference.

An easy way to make use of all the information provided is to average the signed distance for each pair to derive a metric for each item. For example, the tire appears twice, with respective distances of +3 and +7. It can therefore be assigned an average distance of +5. The sandwich also appears twice with signed distances of -5 and -7, giving an average of -6. Applying this logic across the entire training set yields a set of average distances that order the objects in a way that is close to their true ranks. If participants employ this strategy, they should make the most errors on items E and F. Looking at the data in Fig. 2, it appears that this may be the case.

It should be noted that P's are also given the following ordinal relations:

C>H, F>G, D>G, B>F, C>E, B>E, A>D and A>H. From these, one can infer that B>G, A>G. Therefore, one might expect higher accuracy on these pairs than on other test pairs.

**Reply:** Thank you for raising the alternative models. We have explicitly tested them and compared the results with our empirical observations.

About the distance-averaging model, we confirm the reviewer's observation that pairs B-C and E-F showed the highest error rates (ranking 1st and 4th respectively among all 28 pairs). Following the reviewer's suggestion, we implemented a distance-averaging model in which each item's value is calculated as the average of its signed distances across the learned pairs, and choices are generated via a softmax function. This model successfully captures the grand average effect, including both distance effects and serial position effects, in our data, consistent with the reviewer's intuition. Notably, the standard Q-learning yields similar predictions, as it essentially implements the distance-averaging computation with trial-by-trial decay.

Critically, however, the distance-averaging account predicts that participants would perform similar averaging computations and therefore display similar errors—resulting in unimodal accuracy distributions centered around intermediate values. This prediction is inconsistent with our data. We observe clearly bimodal accuracy distributions (Figure 2d): for difficult pairs, some participants are consistently correct while others are consistently wrong. Empirically, when fitting the distance-averaging model to individual participants (matching observed accuracies), it produces unimodal distributions (Figure R1f, grey cells) rather than the bimodal patterns observed in our data (Figure 2f, green cells).

Regarding transitivity-based inference, we examined whether inference via single-step transitivity (e.g., B>G from B>F and F>G) showed enhanced accuracy. The reviewer notes that B>G and A>G should show higher accuracy than other pairs. We found mixed evidence: when controlling for symbolic distance, B>G (94.7%) showed lower accuracy than C>H (98.7%) and A>F(96.5%), while A>G (96.5%) showed higher accuracy than B>H (94.7%), but both also showed similar accuracy to other same-distance pairs. We further tested whether transitive distance (number of logical inference steps) predicted accuracy beyond symbolic distance. While transitive distance showed significant effects, this effect disappeared after controlling for observed versus inferred pairs, suggesting that it primarily reflects direct learning rather than transitive inference (linear mixed-effect model; *F*(1, 1463.00)= 0.17, *p* = 0.68).

Most importantly, neither models can explain or address our central results – participants make globally self-consistent errors. Despite making errors on specific pairs, 92.8% of participants exhibited no circular triads (Figure 3b). If errors arose from independent failures on individual pairs (either due to imperfect distance-averaging or incomplete extraction of relational information), we would expect uncorrelated errors across pairs, leading to circular triads. Instead, the remarkably low proportion of circular triad rates indicate that participants actively construct coherent global rankings that preserve internal consistency.

These new analyses have been incorporated into the revised manuscript, including: (1) comparison of distance-averaging model predictions with empirical accuracy distributions (S5 Fig), (2) analyses of transitive distance effects controlling for symbolic distance and observed versus inferred pairs (S3 Fig c), and (3) expanded Discussion explaining coherence-seeking mechanisms alongside distance-based and transitivity-based accounts (Discussion L345-360).

Discussion L345-360

Our findings largely replicate classical phenomena in transitive inference at the group level, including the canonical serial position and symbolic distance effects—hallmark signatures of relational learning observed across species and paradigms (Bryant & Trabasso, 1971; Vasconcelos, 2008). Notably, these effects emerged despite a fundamental departure from traditional designs: whereas classical paradigms rely on extensive training with adjacent pairs and continuous feedback (Ciranka et al., 2022; Jensen et al., 2015), our participants received only brief few-shot exposure to largely non-adjacent pairs, followed by testing without feedback. Importantly, these group-level effects could also be well captured by classical computational models based on incremental value updating mechanisms (Ciranka et al., 2022; Jensen et al., 2015; Vasconcelos, 2008; Wynne, 1995).

Beyond replicating canonical effects, our results substantially extend previous work on transitive inference by revealing stable, self-consistent, yet idiosyncratic ranking patterns at the individual level. These individual-level structures cannot be readily explained by classical value-updating models, highlighting a dissociation between group-level regularities and individual-level inference. This divergence suggests that incremental value updating alone is insufficient to capture the full nature of relational inference, particularly under few-shot conditions. Rather than passively accumulating evidence and updating items independently, the observed global self-consistency across local pairs points to the active imposition of a relational schema during learning. This *constructive ranking account* aligns with theoretical frameworks emphasizing structural priors (Kemp & Tenenbaum, 2008; Tenenbaum et al., 2011) and geometry-based representations of ranking (Di Antonio et al., 2024; Mannella & Pezzulo, 2024; Nelli et al., 2023; Wen et al., 2025). From this perspective, relational learning does not merely tune local values, but involves the construction of a global relational structure that constrains and supports internal consistency within each individual.

Figure R1. Distance averaging model prediction. Conventions follow Figure 1 and Figure 2.

Overall, it is critical to clearly communicate the rationale for the experimental design and provide a correct analysis of whether there is enough information for P's to learn the entire list order if they were able to use all the information presented.

**Reply:** We are grateful to the reviewer for these critiques and suggestions, which have substantially improved the clarity of the manuscript. In response, we have extensively revised the Introduction to more explicitly and logically situate our rationales and hypotheses within the framework of few-shot learning, emphasizing the inherent cognitive resource limitations and the role of inductive biases in relational inference under sparse samples. Moreover, we have incorporated new analyses and expanded discussions in the revised manuscript to strengthen our central conclusion—active construction, as a form of inductive bias in ranking, accounts for the observed idiosyncratic ranking patterns across individuals and cannot be readily explained by current computational models based on independent value representation.

We believe this revision has led to a clearer articulation of the study’s rationale and a more precise interpretation of our findings. We thank the reviewer for raising these concerns, which has fundamentally strengthened the manuscript.

The second set of concerns has to do with the MEG results.

A simpler way to describe the MEG results is that they might reflect the difference between a visual comparison and an abstract comparison. The interpretation that MEG reflects an internal ranking needs to be explained more clearly.

**Reply:** Thank you for the question. To address whether the two task conditions performed on identical images simply reflect visual versus abstract comparison, we conducted a new analysis on the MEG data, by adding a visual similarity model based on pixel-level image similarity into the RSA.

As shown below, the visual similarity neural effect emerged early in both the pre-learning and post-learning phases (165-290 ms), supporting task-independent, stimulus-driven visual feature processing. In contrast, the real-world size rank effect appeared at an intermediate latency (245-425 ms) in both phases, suggesting that it reflects innate semantic knowledge rather than low-level perceptual features. Critically, the newly formed ranking effect emerged substantially later (685-775ms) and exclusively in the post-learning phase. This clear temporal dissociation—occurring approximately 500ms after the visual similarity effect—provides strong evidence that the ranking effects cannot be attributed to visual comparisons.

Taken together, the temporal progression from visual features to innate semantic knowledge and to abstract structure support that the neural representation of subjective ranking reflects participants' actively constructed internal rankings rather than stimulus-driven visual processing.

These new analyses and results have been incorporated into the Results section (L284-314 and Figure 5).

Figure R2. Distinct time courses for visual similarity, real-world size, and newly acquired ranking effects in MEG representational similarity analysis.

Results L284-314:

Three model representational dissimilarity matrices (RDMs) were constructed (see Fig. S6), capturing low-level visual features, real-world size rankings, and subjective rankings derived from individual behavioral data, respectively. At each time point, we performed multiple linear regression analyses in which each participant’s neural RDM served as the dependent variable and the three model RDMs were entered simultaneously as predictors (Fig 5a). This procedure yielded participant-specific β coefficients that quantified the strength of neural encoding for each relational structure. For real-world size, we averaged the corresponding β coefficients across the pre- and post-learning phases, reflecting its persistent representation as innate knowledge. Critically, for subjective ranking, we computed the difference between post- and pre-learning coefficients, β(post) – β(pre), to isolate neural reorganization specifically attributable to few-shot learning. This controls for pre-existing alignment between intrinsic neural geometry and the to-be-learned structure, ensuring that the observed effects reflect learning-induced changes rather than baseline correlations.

Results revealed temporally and spatially dissociated neural signatures. Pixel-level visual similarity emerged early (165-290 ms) in both pre- and post-learning phases (Fig 5b; cluster-based permutation test, p=0.001), confirming stimulus-driven processing independent of tasks. Real-world size representations appeared at intermediate latencies (240-480 ms) with comparable strength across both phases (Fig 5c; cluster-based permutation test, 240-480 ms, p = 0.001; 710-1015 ms, p = 0.001). Crucially, subjective ranking representations emerged substantially later (685-775 ms, p = 0.041) and occurred only during post-learning (Fig 5d). A permutation test, shuffling subjective ranking RDM across subjects, confirmed the subject-specific nature of global rankings—neural responses aligned with each subject’s own ranking, not with others' (Fig 5f; *p* = 0.01). Sensor-level searchlight analysis revealed distinct spatial distributions: low-level features in right parieto-occipital cortex, real-world size at posterior and lateral sensors, and subjective ranking localized to mid-parietal regions (Fig 5e). Sensor-level searchlight analysis revealed distinct spatial distributions: low-level features were localized to right parieto-occipital sensors, real-world size representations to posterior and lateral sensors, and subjective ranking to mid-parietal sensors (Fig. 5e).

Why was representational dissimilarity used? How does it produce a representation of rank? It is not clear exactly how abstract rank was decoded.

**Reply:** Thank you for the question, and we apologize for not sufficiently explaining the rationale of the RSA in the original manuscript.

Specifically, in using RSA to decode ranking information, we hypothesize that the multivariate neural response similarity between each pair of images is proportional to their rank distance. In other words, image pairs that are closer in rank should elicit more similar neural responses, whereas pairs that are far apart in ranks should elicit less similar response. Based on this logic, we construct model Representational Dissimilarity Matrices (RDMs) for subject ranking in each participant based on their behavioral outcomes, and regress the rank RDM against the neural dissimilarity response calculated from the MEG data. The significant peaks in the RSA results indicate that, at those time points, the pattern of neural response across images could be well explained by the rank-based model RDM, indexing the neural representation of ranking information.

The same framework allows us to construct multiple model RDMs based on different relational structures, including subjective ranking, real-world size, and pixel-level visual similarity, and to regress them against the same MEG data. The RSA approach enables us to examine the respective neural representations and their temporal evolution in a unified manner.

We have now added this rationale for RSA to the Methods (L995-998, L1012-1022).

Methods	L995-998:

To characterize the neural basis of constructed rankings, we employed representational similarity analysis (RSA), which assesses whether neural response patterns reflect hypothesized relational structures by testing whether items that are closer along a given dimension evoke more similar neural activity.

Methods	L1012-1022:

Model RDM construction. Three model representational dissimilarity matrices (RDMs) served as regression predictors. The low-level visual similarity model RDM was identical across all participants and constructed from cosine dissimilarity (1 − cosine similarity) computed across pixels and RGB color channels, capturing bottom-up perceptual differences between stimuli. The size-rank model RDM was also identical across participants, computed from absolute differences in canonical object size rankings (1–8), reflecting shared real-world knowledge about physical object sizes. The subjective-rank model RDMs were uniquely generated for each participant based on their individually reconstructed ranking sequences. This design enabled us to distinguish between neural representations of low-le vel perception, common semantic knowledge, and personalized subjective orderings.

To test the decoder, it should work on multiple lists for the same participant.

**Reply:** We agree with the reviewer that neural representations of ranking reflect abstract relational structure and, in principle, should be independent of specific images and could generalize across different image lists. However, as only one list was used here to probe the neural correlates of abstract ranking, this generalization test could not be directly conducted.

Nevertheless, the RSA results were performed on the same image-evoked neural responses based on different model structures – including the newly learned ranking, real-world size, and low-level visual features – and revealed clearly dissociated spatiotemporal neural patterns. This dissociation suggests that the observed ranking-related neural signature reflects abstract relational information.

Moreover, ranking-related neural representations aligned with each participant's own subjective ranking rather than rankings constructed by other participants, despite identical images and a shared objective ranking across subjects (Figure 5f). This subject-specific alignment provides further evidence that the neural signature captures abstract ranking representations relevant to each individual, independent of the physical images.

There is also a potential performance confound as overall performance was not equal between the different ordering tasks.

**Reply:** The reviewer is correct that the real-world size task, given its innate nature, is likely easier than the newly learned ranking task. Accordingly, the reviewer raised concerns that the observed differences in neural representations between the two ranking tasks may be confounded by task difficulty.

We believe this is unlikely to fully account for the observed dissociation. Notably, the neural representations of the two ranking tasks emerge in largely distinct brain areas: real-world size representations are primarily observed in occipitotemporal sensors, the classical brain region for semantic knowledge, whereas representations of newly learned ranking are expressed in frontoparietal sensors (Figure 5e). This dissociation suggests that the observed neural differences reflect the extraction of different types of information by the brain, rather than a simple effect of task difficulty.

That said, we acknowledge that task difficulty may contribute to certain aspects of the neural differences. For example, the neural representation of real-world size is overall stronger than that of the newly learned ranking (Figure 5b-d), which may partly reflect differences in task difficulty.

## Reviewer #2

Liu et al. presents a study on a transitive inference paradigm with behavioral and MEG measures. The central hypothesis is that few-shot learning from limited information during transitive inference is a constructive process driven by idiosyncratic factors across individuals. Hence, even when providing the same learning pairs during inference, the authors hypothesize that different individuals will have distinct ranking errors that are consistent across repeated testing of the same pairs, reflecting self-consistent global ranking that is incorrect in individualized ways across subjects. The study has several advantages, including using a large sample size for the behavioral study, comparing against null hypothesis based on computational modeling, and using MEG data with RSA to investigate neural correlates. While the overall findings are well presented, several questions impede a clearer contribution to the literature in this study. Hence, I recommend revisions to address these key points before being evaluated for publication.

We appreciate the reviewer’s positive evaluation of our work and the clear and accurate summary of our findings.

MAJOR 1: The main focus of the study is about showing how individuals exhibit idiosyncratic local pair errors under few-shot learning despite the same learning pairs. It is perhaps not very surprising that under a difficult transitive inference setup, individuals will make mistakes, and those mistakes may not agree between individuals. One thread that the authors touched on is how the current models and theories of relational learning overlook this individualized difference. But it is not clear to readers why do the normative models of transitive inference prohibit individualized learning outcomes. Is the main point just that the computational models do not have stochasticity to account for the observed global ranking errors? Perhaps a better explanation of the implication of having or not having individualized learning in the extant models can better contextualize the importance of the current findings.

**Reply:** We thank the reviewer for this thoughtful critique, which helps us clarify our theoretical contribution, and we apologize for not articulating the rationale and key findings sufficiently clear in the original manuscript.

As the reviewer correctly noted, under difficult conditions, individual variation in behavior seems to be largely expected. From this perspective, the observed idiosyncratic rankings across individuals could be simply attributed to stochastic noise during learning. Therefore, our findings might appear unsurprising and readily explainable by extant frameworks.

However, we would like to emphasize that our results reveal a fundamentally different pattern that cannot be readily explained by relevant computational models. As articulated in the revision, the *independent-value account*—which underlies most transitive inference models (e.g., Q-learning, Beta-Q, Betasort)—treats each item as independently represented and updated along a latent value axis, with no constraints imposed by a global ranking structure. Under this framework, stochastic parameters (learning rates, choice noise) capture individual variability by introducing random fluctuations around a shared mean ranking cross subjects. Consequently, errors on individual pairs would arise independently, reflecting trial-by-trial noise in value estimates.

Most importantly, under this account, errors on different pairs should be uncorrelated given their independence, which would naturally lead to circular triad errors (e.g., judging 3 > 4 and 4 > 5, but also judging 3 < 5). In contrast, our results demonstrate that participants make globally self-consistent errors across local pairs, rather than independent local errors, supporting the *constructive ranking account*. Specifically, despite making systematic errors on specific local pairs, 92.8% of participants exhibited globally self-consistent rankings with no circular triads—when participants incorrectly inferred 3rd > 4th and 4th > 5th, they nonetheless maintained the transitive implication 3rd > 5th (Figure 3b). If errors arose from independent noise affecting each pair separately, as predicted by the independent-value account, we would expect substantial numbers of circular triads reflecting uncorrelated local mistakes. Instead, the remarkably low circular triad rate indicates that participants actively construct coherent global rankings that preserve internal consistency across all local relations. In other words, local pair errors are not isolated distortions but are constrained by each individual's subjectively constructed global ranking structure. This self-consistent error pattern cannot be reproduced by simply adding stochasticity to independent-value models.

Therefore, our results reveal a mechanism largely absent from current frameworks: under few-shot learning with computation resource limitations, the human brain actively imposes global relational structure to maintain internal coherence, even at the cost of making systematic errors on specific local comparisons. Rather than passively accumulating independent pairwise evidence, participants construct unified ranking schemas guided by inductive biases—internal constraints that shape hypothesis space and enforce transitivity. This constructive process reflects the active, generative nature of human relational learning under sparse samples and cognitive resource limitations.

We are grateful to the reviewer for raising this important question. We have added clarifications and discussion in the revised manuscript explaining why stochastic extensions of existing models cannot account for our central findings (L342-357, L371-386).

Discussion L334-344

Beyond replicating canonical effects, our results substantially extend previous work on transitive inference by revealing stable, self-consistent, yet idiosyncratic ranking patterns at the individual level. These individual-level structures cannot be readily explained by classical value-updating models, highlighting a dissociation between group-level regularities and individual-level inference. This divergence suggests that incremental value updating alone is insufficient to capture the full nature of relational inference, particularly under few-shot conditions. Rather than passively accumulating evidence and updating items independently, the observed global self-consistency across local pairs points to the active imposition of a relational schema during learning. This *constructive ranking account* aligns with theoretical frameworks emphasizing structural priors (Kemp & Tenenbaum, 2008; Tenenbaum et al., 2011) and geometry-based representations of ranking (Di Antonio et al., 2024; Mannella & Pezzulo, 2024; Nelli et al., 2023; Wen et al., 2025). From this perspective, relational learning does not merely tune local values, but involves the construction of a global relational structure that constrains and supports internal consistency within each individual.

Discussion L371-386

One might argue that the observed idiosyncratic rankings across individuals simply reflect stochastic noise during learning, particularly under challenging conditions such as few-shot learning. However, this explanation cannot account for the pronounced self-consistency observed within individuals. In fact, participants exhibited remarkably few circular triad errors in local pairwise comparisons, indicating that local errors are not isolated noisy distortions but are instead constrained by each individual’s subjectively constructed global ranking. In other words, the observed pattern of self-consistent errors cannot be explained by merely adding stochasticity to traditional independent-value models. Rather, our results point to a mechanism largely absent from current frameworks – the human brain actively imposes a global relational structure to maintain internal coherence, even at the cost of making systematic errors on specific local comparisons. Instead of passively accumulating independent pairwise evidence, participants construct unified ranking schemas guided by inductive biases—internal constraints that shape the hypothesis space and enforce transitivity. This constructive process underscores the active, generative nature of human relational learning under sparse samples and cognitive resource limitations.

In addition, how do the current results agree with previous studies showing the success of transitive inference models in explaining participants behaviors? If such strong inter-individual variability also existed in previous studies, one would have expected poor model fitting. Figure 3 e. shows that even though subjects make local errors, the overall performance is quite good and there is certainly a correlation with the ground-truth global ranking across subjects (darker color to the right of bars).

**Reply:** Thank you for the question. Our results (Figure 1e–g) are indeed highly compatible with previous studies and can be well accounted for by established computational models (e.g., Q-learning, Beta-Q, Betasort) at the *group level*, capturing canonical effects such as overall accuracy, serial position effects, and symbolic distance effects. In this sense, we fully agree that these models successfully explain average performance patterns across participants.

However, the key distinction—and our central contribution—is that these models do not account for the internal structure of local pair errors at the individual level. While they reproduce group-level accuracy, they fail to explain the observed self-consistent, idiosyncratic local errors within individuals.

As correctly noted by the reviewer, Figure 3e shows overall good performance across subjects, indicating that subjective rankings do not deviate substantially from the ground truth. Importantly, this observation strengthens rather than weakens our argument. Participants did not just perform randomly or construct arbitrary rankings; instead, most individuals largely preserved the global structure of the true ranking while introducing systematic and internally consistent local deviations. Such structured individual-specific errors are not readily captured by existing models, as demonstrated by our modeling analyses (S6 Fig).

We have clarified this point and added relevant results and discussion in the revised manuscript (L331-341).

Results L141-146

To further validate these canonical effects, we fit a standard Q-learning model (Jensen et al., 2015; Ciranka et al., 2022) to participants’ trial-by-trial choices (see Methods). As shown in Fig 1h–j, the model successfully reproduced both the serial position and symbolic distance effects, confirming that our paradigm captures relational inference phenomena consistent with prior work (see S4 Fig bc, for Beta-Q and Betasort model fits).

Discussion L331-341

Our findings largely replicate classical phenomena in transitive inference at the group level, including the canonical serial position and symbolic distance effects—hallmark signatures of relational learning observed across species and paradigms (Bryant & Trabasso, 1971; Vasconcelos, 2008). Notably, these effects emerged despite a fundamental departure from traditional designs: whereas classical paradigms rely on extensive training with adjacent pairs and continuous feedback (Ciranka et al., 2022; Jensen et al., 2015), our participants received only brief few-shot exposure to largely non-adjacent pairs, followed by testing without feedback. Importantly, these group-level effects could also be well captured by classical computational models based on incremental value updating mechanisms (Ciranka et al., 2022; Jensen et al., 2015; Vasconcelos, 2008; Wynne, 1995).

The authors discussed differences in the paradigm from standard transitive inference, so how much of the observed idiosyncratic ranking just due to a harder paradigm where subjects? Or perhaps the same level of variability has always existed but just did not impact the general agreement between model-based relational learning and subject performance? Some level of variability must be expected in such task, just like response time is not identical across subjects. So how should readers appreciate the effect size of the variability reported here?

These should be better described and discussed - otherwise it is unclear why the current results are meaningful to the literature on relational learning beyond just "noise".

**Reply:** We appreciate the important question. As explained above, our results reveal a pattern that is largely absent from the current understanding of ranking and transitive inference: participants make globally self-consistent errors across all pairs, indicating that they actively construct coherent global rankings with idiosyncratic internal consistency. In other words, local pairwise errors are constrained by each participant’s subjectively constructed global ranking, rather than arising independently across pairs.

As the reviewer suggests, we believe that such subjective rankings are most likely to emerge under challenging conditions, when available information is sparse and computation resource is limited, as in the few-shot learning here. Under these conditions, the brain appears to actively impose *biased* structure to preserve internal consistency across pairs, even at the cost of making systematic errors on specific local comparisons. This pattern is consistent with theoretical and empirical work on inductive biases under sparse data regimes (Tenenbaum et al., Science, 2011; Lieder & Griffiths, 2020; Feldman, 2000).

The reviewer further raises the possibility that the observed effects may simply reflect general inter-subject variability and asks whether the effect size can be quantified. To address this concern, we conducted systematic quantitative comparisons between the empirical data and multiple computational models of transitive inference. Specifically, we fitted Q-learning, Beta-Q, and Betasort models to individual participants, matching their observed accuracy levels. We additionally tested the distance-averaging model suggested by Reviewer 1, as well as a “correct-ranking” baseline model. Despite achieving comparable accuracy to human participants, all models produced substantially lower inter-subject variability in inferred rankings than observed empirically. In particular, inter-subject ranking similarity (Kendall’s tau) was consistently higher in all model simulations than in the empirical data (Figure R3), indicating that the degree of individual divergence in subjective rankings far exceeds model predictions, even when task difficulty and average performance are well matched.

Taken together, the theoretical significance of our findings lies in revealing how the brain responds when relational inference exceeds cognitive capacity, as in few-shot learning. Rather than producing uncorrelated, stochastic errors—as assumed by most existing models—human learners appear to engage coherence-seeking mechanisms that enforce internal consistency across local relations, even at the expense of making systematic local errors. This coherence-seeking behavior reflects an inductive bias that shapes relational learning under sparse data conditions.

We thank the reviewer for these thoughtful critiques, which have substantially improved the clarity of the manuscript. In response, we have extensively revised the Introduction (L67-93) to more explicitly motivate our hypotheses within a few-shot learning framework, emphasizing cognitive resource limitations and inductive biases in relational inference. We have also added new modeling analyses in the Results.

Figure R3. Inter-subject ranking similarity: empirical data vs. model simulations.

Introduction L67-93

Because ranking involves stochastic relational inference under uncertainty (Lake et al., 2015; Tenenbaum et al., 2011), we hypothesized that inductive biases are engaged during few-shot learning of local pairwise relations. Under sparse evidence and limited computational resources, relational inference is therefore not a passive accumulation of evidence but is shaped by the active construction of a global ranking structure within each individual—an account we refer to as the *constructive ranking account* (Fig 1a, right). According to this account, individuals actively construct a unified and idiosyncratic global ranking from few-shot experience, which serves as an inductive bias to constrain learning of local pairs. As a result, even when exposed to identical local pairwise information, individuals tend to form stable and self-consistent, yet idiosyncratic, global rankings that deviate from the ground truth. This account contrasts sharply with the classical *independent-value account* (Fig 1a, left), previously used to explain transitive inference (Ciranka et al., 2022; Jensen et al., 2015). Under this account, each item is represented and updated independently as a point on a latent value axis, with no constraints imposed by a global ranking structure.

A classical framework for studying relational learning is transitive inference (Acuna & Sanes, 2002; Bryant & Trabasso, 1971; Piaget, 1923; Vasconcelos, 2008), in which relationships between non-adjacent items (e.g., A < C) are inferred from relationships between adjacent items (e.g., A < B, B < C). A related phenomenon, list linking (Nelli et al., 2023; Treichler & Van Tilburg, 1996; Wei et al., 2014), occurs when two independently learned sequences are rapidly integrated into a single ordered list after learning a single bridging relation. Numerous computational and neural network models have been proposed to account for such relational learning (Ciranka et al., 2022; Delius & Siemann, 1998; Jensen et al., 2015; Mannella & Pezzulo, 2024; Wynne, 1995; Kay et al., 2024; Miconi & Kay, 2025; Mitchell, 2007; Nelli et al., 2023). Here, we adapted the transitive inference paradigm within a few-shot learning framework to directly examine the role of inductive bias in relational inference.

Results L182-189

Notably, Q-learning simulations with matched accuracy failed to reproduce the observed bimodal patterns. Simulated difficult pairs yielded unimodal distributions (grey cells, Fig 2f versus green cells, Fig 2d), and simulated participants showed low error consistency with only 7 of 69 exceeding the 80% threshold (Fig 2g). Beta-Q and Betasort models showed similar results (Fig S4). Therefore, these computational models based on independent-value account, though successful at capturing group-level performance as shown in Fig 1, cannot account for the polarized, consistent error structure in individuals.

Results L223-231

Furthermore, computational models based on the independent-value account failed to reproduce the observed self-consistency. Specifically, we evaluated five models: three learning-based models that update item values from observed pairs (Q-learning with point estimates, BetaQ with distributional values, and Betasort with cross-item inference), one baseline model that averages presented distances (distance-averaging), and one oracle model using ground-truth values (correct-ranking). In contrast, model simulations predominantly produced either correct rankings or self-inconsistent errors, rather than the self-consistent but incorrect rankings observed in most of human participants (Fig 3b, right; S6 Fig).

Finally, we reconstructed each participant’s subjective ranking based on their own 28 pairwise judgments using the HodgeRank method (see Methods; example subject shown in Fig 3c). This analysis revealed individualized global rankings that deviated from the ground-truth ordering, resulting in low inter-individual similarity (Fig 3e). In contrast, simulations of independent-value computational models produced substantially higher inter-subject similarity (Fig 3d; Q-learning, two-sample t-test, t = −66.77, p < 0.001; see S6 Fig for results from other models), indicating that the classical models predict convergence toward a shared ranking rather than the observed idiosyncratic rankings.

MAJOR 2: One aspect of the learning phase that concerns me is the order of learning pairs. The methods section stated that the ranking sequence of each participant was randomly shuffled in the learning phase. So even though the exact eight pairs used in few-shot learning is random, but the order with which they are presented is different across subjects. Then a major question is how much the ordering of the learning pair presentation influenced the idiosyncratic learning across subjects. Had subjects been given the same ordering of the same learning pairs, making it truly identical, would subjects show more consistency in constructed global ranking? This seems to be a major confound given the central claim is about individualized global ranking and learning outcomes.

**Reply:** Thank you for raising this important concern regarding the extent to which the ordering of learning-pair presentation might influence idiosyncratic learning across participants. If presentation order were a primary driver of individualized rankings, we would expect participants exposed to more similar sequences of local pairs during learning to construct more similar subjective global rankings.

To directly test this possibility, we conducted a control analysis quantifying, for each pair of participants, both learning sequence similarity and ranking similarity. Learning sequence similarity was measured using a normalized Levenshtein distance (1 minus edit distance divided by sequence length), and ranking similarity was quantified using Kendall’s tau. Under the presentation-order account, higher sequence similarity should predict higher similarity in subjective rankings across participants. As shown below (Figure R5), we found no significant correlation between learning sequence similarity and ranking similarity (r = -0.015, p = 0.423). This null relationship remained when restricting the analysis to specific learning blocks to examine potential primacy or recency effects (Block 1: r = -0.012, p = 0.516; Block 2: r = 0.006, p = 0.758; Block 3: r = -0.018, p = 0.322; Block 4: r = -0.005, p = 0.798).

To further test whether learning sequence order drives inter-individual variability in subjective rankings, we conducted a supplemental between-group experiment (N=40). Participants were randomly assigned to one of two groups (N=20 each), with each group exposed to a different learning sequence during the few-shot learning phase. Critically, all other aspects of the paradigm remained identical to the original behavioral experiment. Under the presentation-order account, subjective ranking similarity within the same group should be higher than between-group similarity, as participants with same experience should have similar construction process. However, we found no significant difference in inter-subject ranking similarity between within-group pairs and between-group pairs (Figure R6; independent samples *t*-test: *t* = 0.02, *p* = 0.99).

Together, these results provide no evidence that differences in presentation order are a major source of the observed inter-individual differences in global rankings.

While we cannot entirely rule out a contributory role of presentation order, importantly, this factor does not account for our central finding: participants reliably maintain globally coherent yet individualized rankings despite substantial variability in learning sequences. Such robustness to input order variability is consistent with a coherence-seeking inductive bias, whereby the brain actively imposes a stable global structure under sparse and noisy learning conditions.

We have incorporated the corresponding analysis and new experiments in the Discussion (L417-420) and supplementary materials (S8 Fig and S9 Fig).

Figure R5. Inter-subject ranking similarity: empirical data vs. model simulations.

Figure R6. Inter-subject within each presentation group and between two presentation groups.

Discussion (L417-420)

Some research indicates that learning process may influence the learning results (Bengio, 2009), yet both correlational analyses and a controlled between-group experiment found no evidence that learning sequence order drives the observed inter-individual variability in subjective rankings (S8 Fig and S9 Fig).

MAJOR 3: The MEG task differs from the behavioral-only paradigm in an important way: the pre-learning biased subjects to evaluate the images on a common and accessible semantic knowledge dimension: size. Then the few-shot learning requires subjects to unlearn and overcome this pre-learning phase bias in order to learn the actual arbitrary global ranking among the objects. A comparison of the level of inter-individual variability as well as within-subject consistency in errors across the MEG and behavioral tasks will be helpful. Such result can help readers appreciate the effect size of the reported variability results on the basis of how much a pre-existing semantic knowledge influences the relational learning. In real life, people often have variable semantic knowledge and intrinsic feature values, so how much is "identical local learning" really relevant in ecological settings is unclear.

**Reply:** Thank you for the insightful question. To directly address the reviewer’s concern, we conducted systematic comparisons of inter-individual variability and within-subject consistency between the behavioral task and the post-learning phase of the MEG experiment.

To directly address this concern, we conducted systematic comparisons between the behavioral task and the post-learning phase of the MEG experiment (Figure R7). Critically, inter-subject ranking similarity in the MEG post-learning phase was significantly lower than that observed in the behavioral experiment (independent t-test, t_3275_ = 8.17, p < 0.001), indicating that participants did not converge toward a shared ranking structure influenced by pre-existing real-world size knowledge. Within-subject error consistency remained comparable across behavioral and MEG post-learning phases (Behavioral: 0.87; MEG: 0.89; t = -0.71, p = 0.48), as did the proportion of participants exhibiting globally self-consistent rankings (MEG: 26/26, 100%; Behavioral: 64/69, 92.8%). Most importantly, within-subject correlations between real-world size rankings and individually reconstructed subjective rankings were essentially zero (mean r = 0.03, 95% CI [-0.06, 0.13]), providing direct evidence that the constructed rankings were independent of pre-existing object size knowledge (Results L271-273).

We further emphasize that the pre-learning phase was not intended as a learning manipulation requiring participants to “unlearn” size knowledge, but rather as a baseline measurement without feedback. This design follows established practices in prior work (Nelli et al., *Neuron*, 2023) and serves two key purposes: (1) to establish within-subject baseline neural geometry prior to few-shot learning, and (2) to enable a subtraction analysis, β₂(post) − β₂(pre), that isolates learning-specific neural reorganization while controlling for stimulus-driven effects and pre-existing individual differences. Consistent with this rationale, we observed a significant post–pre difference (Figure 5d, p = 0.041), together with nonsignificant correlations between real-world size and subjective rankings, providing empirical validation that this control successfully isolates learning-induced reorganization independent of baseline variability.

The reviewer correctly notes that individuals in the real world possess variable prior knowledge. Here we deliberately used identical arbitrary inputs to exclude such variability and to demonstrate that participants construct idiosyncratic yet coherent rankings that are uncorrelated with pre-existing knowledge. In more naturalistic settings, where both inputs and priors vary, such constructive mechanisms would be expected to play an even more prominent role.

We have clarified these points and added relevant detail in Results (L269-273, L251-264).

Figure R7. Comparison of inter-individual variability and within-subject consistency between behavioral and MEG post-learning phase.

Results L269-273

Behavioral patterns in the MEG cohort closely matched those observed in the preregistered behavioral experiments (S7 Fig). Furthermore, the constructed rankings were independent of real-world size knowledge (mean r = 0.03, 95% CI [−0.06, 0.13]; S10 Fig), indicating that the pre-existing semantic structure did not impact learned rankings.

Results L251-264

The MEG experiment comprised three phases. In the pre-learning phase, participants performed a sequential comparison task on eight images, judging whether each item was larger or smaller in real-world size than the preceding item (5 blocks × 81 images, yielding 80 pairwise judgments per block; no feedback provided). Next, participants completed the few-shot learning phase, identical to that used in the preregistered behavioral experiment. In the post-learning phase, participants performed the same sequential comparison task as in the pre-learning phase, but based on the newly learned ranking. Thus, the pre- and post-learning phases employed identical stimuli, timing, and task demands, differing only in the underlying ranking rule. This design offers two advantages. First, the pre-learning phase served as a within-subject baseline, capturing stimulus-driven neural responses and pre-existing representational structure. Second, contrasting post-learning with pre-learning activity isolated neural reorganization specifically attributable to newly constructed rankings, while controlling for stimulus properties and individual differences in baseline activity.

MAJOR 4: The beta weights reported in Figure 4 should be better unpacked. Model RDM 2 should not have any meaningful beta weights during pre-learning, since the subjects haven't been exposed to the arbitrary ranking yet, it doesn't make sense that it should explain any neural RDM pattern. Because of that, it is strange to subtract post-learning from pre-learning for the subject-specific rank - why not just use the post-learning alone? Also, g and i have similar scales. But one would have expected a summation of two beta weights to be larger than the difference between two beta weights. Therefore, these results should be better presented/explained.

**Reply:** We appreciate these questions. The reviewer first raised the concern that Model RDM 2 should not yield meaningful beta weights during the pre-learning phase, and questioned why we subtract pre-learning from post-learning rather than analyzing post-learning alone.

We would like to clarify that the pre-learning beta weights for Model RDM 2 are indeed meaningful and serve as a critical within-subject baseline. Importantly, beta weights estimated in the post-learning phase reflect two components: (1) learning-induced neural reorganization, and (2) pre-existing alignment between intrinsic neural representational geometry and the to-be-learned structure. Without subtracting the pre-learning baseline, it is not possible to dissociate these two contributions. The subtraction design therefore removes this confound and allows us to isolate the neural reorganization specifically attributable to learning, rather than spurious correlations present prior to learning.

Regarding the second concern about scale differences, we apologize for a presentation error in the original manuscript. Figure 5c (previously Figure 4g) was intended to show the sum of beta coefficients across pre-learning and post-learning phases for the real-world size rank model. However, the original figure mistakenly displayed only the pre-learning results. We have now corrected this panel to show the sum values, which do not alter our conclusions. For clarity, we also plot the raw beta coefficients below (Figure R6).

We have added rationales for using the pre-learning phase as a baseline (Results L289-297), corrected the figure accordingly (Figure 5c), and—following Reviewer 3’s suggestion—added the raw beta coefficients for both pre-learning and post-learning phases as a new panel in Figure 5 (b) for clarity.

Figure R6. Raw beta coefficients for both pre-learning and post-learning phases.

Results L289-297

This procedure yielded participant-specific β coefficients that quantified the strength of neural encoding for each relational structure. For real-world size, we averaged the corresponding β coefficients across the pre- and post-learning phases, reflecting its persistent representation as innate knowledge. Critically, for subjective ranking, we computed the difference between post- and pre-learning coefficients, β(post) – β(pre), to isolate neural reorganization specifically attributable to few-shot learning. This controls for pre-existing alignment between intrinsic neural geometry and the to-be-learned structure, ensuring that the observed effects reflect learning-induced changes rather than baseline correlations.

MINOR 1: The authors keep using the term "non-adjacent" pairs throughout the text. But in Figure 1 a and b, isn't the violin and apple pair adjacent rather than "non-adjacent"?

**Reply:** We acknowledge that our use of "non-adjacent" was imprecise and have removed them.

## Reviewer #3

The current study aimed to better understand how humans form rankings of items. For this, they used a few shot learning approach of an 8-item ranking. Participants saw four blocks of eight pairs from the possible 28 pairs; each pair was accompanied with a bar to depict the item value allowing to learn the relative value of each pair with an individual offset from zero without informational value. During testing, each possible pair was shown and participants decided which was worth more. This was done ten times in blocks. In an additional experiment, participants performed a similar task in the MEG. Here the testing occurred in a long sequence and a judgement was made for each item and the preceding item. The behavioral data mainly showed that participants made individual mistakes, i.e., there was no common pattern in the errors. The MEG data mainly showed that representations of the learned ranking can be observed after roughly 700 ms. In general, the study is performed with high methodological rigor, although I do not find the conclusions to be overly novel. When reading, I had some specific concerns.

We thank the reviewer for this thoughtful critique, which helps us clarify our theoretical contribution, and we apologize for not articulating the rationale and key findings sufficiently clear in the original manuscript.

1. I am unsure that I understood the modeling correctly. The authors use Q-learning modeling and flavors thereof (they give no rational for the specific models). Learning was without feedback or choice (observing value pairs) and testing was without feedback. It is unclear to me how the values are learned by the model, if no "prediction error" occurs. Can the authors explain more clearly, how values were updated? The paper states that observed absolute values were non-informative and only the difference matters. It would seem that value-updating can become additionally distorted by this. Maybe the authors can provide a source, where the rationale of this type of modeling is validated. Additionally, I would like to know if the authors would argue that value learning by explicitly showing value pairs is learned by reinforcement learning mechanisms.

**Reply:** We apologize for not making the rationale for the Q-learning–based modeling sufficiently clear in the original manuscript.

First, as correctly noted by the reviewer, our paradigm differs from classical reinforcement learning tasks in that participants did not receive explicit feedback and did not make overt choices during learning. However, prediction errors can nevertheless arise naturally in our task. Each item appears repeatedly across multiple pairings, allowing participants to accumulate an implicit value estimate for each item over time. When an item is encountered in a new pair, participants can generate a prediction about its relative value based on prior exposures. The discrepancy between this predicted relative value and the currently observed relational distance constitutes a prediction error, which can drive incremental updating of item values. This learning logic is explicitly implemented in our model (Equations 3–4) and closely parallels the update rules used in Ciranka et al. (2022; Equations 3a–3b) during their testing phase. The key principle is that value learning proceeds through iterative refinement as items are repeatedly encountered in different relational contexts, with prediction error distributed across both items in a pair. Therefore, the reviewer is correct that value learning by explicitly showing value pairs is learned by reinforcement learning mechanisms.

More broadly, learning in the absence of explicit action–outcome feedback has been widely modeled within both reinforcement learning and self-supervised learning frameworks, particularly for relational and structural knowledge. For example, Gläscher et al. (2010, *Neuron*) demonstrated dissociable neural systems for reward prediction errors and state prediction errors, showing that learning can proceed via prediction of state transitions even without reward feedback. Similarly, Stachenfeld et al. (2017, *Nature Neuroscience*) formalized how the hippocampus acquires predictive representations of environmental structure through successor representations, which are learned from state-to-state transitions rather than action–reward contingencies. In the domain of transitive inference, value-updating frameworks have been applied specifically to relational learning contexts in which information accumulates across repeated exposures (e.g., Jensen et al., 2015; Ciranka et al., 2022).

Accordingly, we did not develop these models de novo. Instead, we adapted established computational frameworks from the transitive inference literature, drawing in particular on Jensen et al. (2015) and Ciranka et al. (2022), which represent the prevailing theoretical accounts in this field. Our adaptation involved implementing the same value-updating logic in a supervised format appropriate for our paradigm, in which relational distance is directly presented rather than inferred from binary feedback.

Critically, these models successfully capture the canonical behavioral signatures of transitive inference in our data. As shown in revised Figures 1h–j and reported in the Results, the models accurately reproduce overall accuracy, the serial position effect (enhanced performance for extreme items), and the symbolic distance effect (reduced accuracy for closely ranked pairs). These results indicate that the models provide valid computational accounts of standard relational learning mechanisms.

However and notably, despite capturing these hallmark effects, the models fail to account for our central finding: the presence of globally self-consistent yet idiosyncratic local errors within individuals. This limitation points to an additional mechanism not captured by existing frameworks. When information is sparse and noisy, as in the few-shot learning regime studied here, human learners appear to actively impose *biased* structure to maintain internal coherence across relations, even at the cost of making systematic local errors. This coherence-seeking behavior reflects an inductive bias that shapes relational inference under sparse data conditions.

We have added clarifications regarding the rationale for the Q-learning model in the revised manuscript (Results L141-148; Results L182-189; Results L223-231; Discussion ).

Results L141-148

To further validate these canonical effects, we fit a standard Q-learning model (Jensen et al., 2015; Ciranka et al., 2022) to participants’ trial-by-trial choices (see Methods). As shown in Fig 1h–j, the model successfully reproduced both the serial position and symbolic distance effects, confirming that our paradigm captures relational inference phenomena consistent with prior work (see S4 Fig bc, for Beta-Q and Betasort model fits). Together, these results indicate that participants infer relational knowledge from few-shot learning of local pairs, which could be well captured by standard computational models at the group level.

Results L182-189

Notably, Q-learning simulations with matched accuracy failed to reproduce the observed bimodal patterns. Simulated difficult pairs yielded unimodal distributions (grey, Fig 2f vs. green, Fig 2d), and simulated participants showed low error consistency with only 7 of 69 exceeding the 80% threshold (Fig 2g). Beta-Q and Betasort models showed similar results (Fig S4). Therefore, the computational models based on independent-value account, though successful at capturing group-level performance (see Fig.1 h–j), cannot account for the polarized, consistent error structure in individuals.

Results L223-231

Furthermore, computational models based on the independent-value account failed to reproduce the observed self-consistency. We evaluated five models: three learning-based models that update item values from observed pairs (Q-learning with point estimates, BetaQ with distributional values, and Betasort with cross-item inference), a baseline model that averages presented distances (distance-averaging; S5 Fig), and a model using ground-truth values (correct-ranking; S5 Fig). All the models predominantly produced correct global rankings accompanied by self-inconsistent local errors, rather than the incorrect global rankings yet self-consistent local errors observed in the majority of human participants (Fig 3b, right; S6 Fig; S11 Fig).

Discussion L338-348

…Importantly, these group-level effects could also be well captured by classical computational models based on incremental value updating mechanisms (Ciranka et al., 2022; Jensen et al., 2015; Vasconcelos, 2008; Wynne, 1995).

Beyond replicating canonical effects, our results substantially extend previous work on transitive inference by revealing stable, self-consistent, yet idiosyncratic ranking patterns at the individual level. These individual-level structures cannot be readily explained by classical value-updating models, highlighting a dissociation between group-level regularities and individual-level inference. This divergence suggests that incremental value updating alone is insufficient to capture the full nature of relational inference, particularly under few-shot conditions…

2. In general, the authors need to be more clear that only the behavioural study was preregistered. I found this quite misleading. In addition, I found the preregistration quite short and hard to understand. Hence, it was challenging to understand where authors deviated from the preregistered analysis plan. I would ask the authors to make this explicit in the manuscript. Additionally, the preregistration states sampling of 40 participants, but nearly twice this amount was recruited! Although this is acknowledged in the manuscript, it is only said that this was done to further validate the findings, but it is not said how authors deal with changes in the stopping rule and resulting alpha inflation!

**Reply:** We thank the reviewer for raising this important methodological concern regarding preregistration, and we apologize for the lack of clarity in our original manuscript. The reviewer noted that the final sample size (*N* = 80) exceeded the preregistered plan (*N* = 40) and expressed concern about potential flexible stopping and alpha inflation.

Our preregistration (<https://osf.io/dpxq4>) specified two core hypotheses to be tested on an initial sample of *N* = 40 participants: (1) Hypothesis I predicted bimodal accuracy distributions across subjects and pairs, tested via Beta distribution fitting to individual participants ($\alpha<1$ and $\beta<1$), and (2) Hypothesis II predicted globally self-consistent rankings within individuals, tested via permutation-based self-consistency analysis. Both preregistered hypotheses were fully confirmed in the initial *N*=40 cohort prior to recruiting any additional participants.

The decision to recruit an additional cohort of 40 participants was made explicitly as an internal replication to strengthen the robustness of the findings, rather than to achieve statistical significance through flexible stopping. When preregistered hypotheses are already confirmed in the initial sample, adding an independent replication does not inflate Type I error; instead, it provides additional protection against false positives by requiring effects to replicate in a new cohort. Consistent with this rationale, we report results for both cohorts separately (S1 Fig and S2 Fig), demonstrating that both independently confirm the preregistered hypotheses. Although we present combined results (*N* = 77) in the main text for narrative clarity, this does not constitute flexible stopping since each cohort meets the preregistered confirmation criteria independently.

In response to the reviewer's concern, we have substantially revised the Methods to include an expanded "Preregistration and Deviations" subsection (L816-841), which now explicitly documents: (1) what was preregistered (*N*=40, two hypotheses with specific analyses), (2) confirmation of both hypotheses in the initial cohort, (3) the independent replication cohort and separate reporting of results (Figure R7 and Figure R8, added as S1 Fig and S2 Fig), (4) one deviation from preregistration (revision of self-consistency coefficient denominator from 56 to 20 based on theoretical maximum), and (5) exploratory analyses conducted beyond preregistered tests (pair-level Beta fitting and error consistency metrics) which provide complementary perspectives without alpha inflation concerns.

We also state now that both pre-registration and replication cohorts in Results (L124-129). Moreover, we now explicitly state that the MEG experiment was a separate, non-preregistered study conducted with an independent cohort (Method L842-843), eliminating any potential confusion about which components were preregistered.

Figure R7. Behavioral results from the pre-registered cohort (N=40).

Figure R8. Behavioral results from the replication cohort (N=37; 3 excluded for below-chance learning performance).

Results L124-129

Notably, our preregistered study specified a target sample size of N = 40. After confirming the preregistered hypotheses in the first cohort (N = 40), we recruited an independent replication cohort (N = 40; three participants excluded due to below-chance accuracy). Because both cohorts exhibited consistent patterns in all preregistered analyses, we report combined results below (N = 77; see S1 Fig and S2 Fig for cohort-specific results).

Methods L816-841

**Preregistration and deviations**

We pre-registered the behavioral experiment on the Open Science Framework prior to data collection (<https://osf.io/dpxq4>). In this pre-registration, we specified our novel behavioral paradigm, target sample size (*N*=40), measured variables, and statistical analysis. Specifically, we preregistered two core hypotheses: Hypothesis I predicted bimodal accuracy distributions across subjects and pairs, tested via Beta distribution fitting to individual participants' accuracy profiles ($\alpha<1$ and $\beta<1$). Hypothesis II predicted globally self-consistent rankings within individuals, tested via permutation-based self-consistency analysis.

After confirming both hypotheses in the pre-registration cohort (*N*=40), we recruited an independent replication cohort (*N*=40; three participants excluded due to below-chance learning performance) to strengthen the robustness of our findings. Although we report combined results (*N*=77) in the main text for narrative clarity, both cohorts independently confirmed both preregistered hypotheses when analyzed separately (S1 Fig and S2 Fig).

Deviation from preregistration. In calculating the self-consistency coefficient (Equation 1), our preregistration used all possible triads ($N_{T}$ = 56) as the denominator. However, subsequent theoretical analysis revealed that for 8 items, the maximum number of circular triads is 20, not 56 (Moran, 1947). We therefore revised the denominator to $N_{T}$ = 20 to accurately reflect the theoretical maximum.

Exploratory analyses. Beyond the pre-registered individual-level Beta distribution fitting (Hypothesis I), we conducted two additional analyses to provide complementary perspectives on error patterns without introducing alpha inflation concerns: (1) pair-level Beta distribution fitting across subjects (Fig 2d vs. 2f), revealing bimodal distributions for difficult pairs at the group level, and (2) error consistency analysis (Fig 2e, 2g), quantifying the proportion of consistently erroneous pairs within individuals.

Methods L842-843

The MEG experiment was a separate, non-preregistered, follow-up study conducted with an independent cohort after the behavioral experiments.

3. When reading the introduction, it was hard to understand the study's rationale and how it advances previous work. It seems intuitively plausible that participants would form idiosyncratic rankings based off their learning experience even if the learning experience is kept constant. This is because the brain's ability to encode information fluctuates constantly, e.g., at the millisecond scale due to ongoing brain oscillations and at the minute scale due to attention allocation (not to mention circadian rhythms etc.). This will automatically lead to individual participants encoding and consolidating some trials and failing to encode and consolidate others with no correlation between subjects. The finding of a bimodal distribution in responses thus seems to result from this well-known fact. I understand that the participants had 4 blocks of learning, but essentially getting rid of this effect would require learning to 100, which defeats the purpose. Additionally, this bimodality emerges mainly for distant unlearned pairs, which are the more difficult inferences to make and here participants seem to be showing a consistent error pattern. Again not surprising or new. Unless I am missing something, this "effect" is common knowledge in the field.

**Reply:** We apologize for not articulating the rationale and key findings with sufficient clarity in the original manuscript. We also thank the reviewer for this thoughtful concern and critique, which has helped us sharpen our theoretical contribution.

As the reviewer and other reviewers correctly note, individual variability in behavior is largely expected, given many contributing factors such as attentional fluctuations, neural variability, task difficulty, and noise in learning. Under this perspective, our findings might indeed appear unsurprising and readily explainable by existing frameworks that treat individual differences as stochastic variation.

However, the critical point of our study does not concern *whether* participants make individual errors on local pairs—which we agree is unsurprising—but *how these local errors relate to one another*. Under independent-value account (Added now in Introduction), each item is represented and updated independently on a latent value axis, with no constraints imposed by a global ranking structure. Consequently, errors on different pairs should be uncorrelated, which would naturally lead to circular triad errors (e.g., judging 3 > 4 and 4 > 5, but also judging 3 < 5). In contrast, our results demonstrate that participants make globally self-consistent errors across local pairs, rather than independent local errors. Despite systematic errors on specific pairs, 92.8% of participants exhibited no circular triads. The remarkably low rate of circular triads indicates that participants actively construct coherent global rankings that preserve idiosyncratic internal consistency across pairs. In other words, local pair errors are constrained by each participant’s subjectively constructed global ranking, rather than arising independently at the local level. As shown in our modeling analyses, this structured error pattern cannot be captured by classical models of transitive inference based on independent value representation.

The reviewer notes that “…the participants had 4 blocks of learning, but essentially getting rid of this effect would require learning to 100, which defeats the purpose”. We agree that the emergence of self-consistent ranking errors is closely related to few-shot learning, where the task is challenging and information is sparse and noisy. Importantly, however, what we find striking is that under these conditions, participants do not produce random or noisy local errors. Instead, following few-shot learning, the brain actively imposes a *biased* global structure to maintain internal consistency across local pairs, even at the cost of making systematic errors on specific comparisons. We propose that this active construction reflects inductive biases in relational inference under sparse samples and cognitive resource limitations—mechanisms that are not yet incorporated in current understanding of transitive inference.

We are grateful to the reviewer for these critiques and suggestions. In response, we have extensively revised the Introduction (L46-93) to more explicitly situate our rationale and hypotheses within a few-shot learning framework, emphasizing cognitive resource limitations and the role of inductive biases in relational inference under sparse conditions. We have also incorporated expanded discussions (L331-357)

in the revised manuscript to strengthen and clarify our central findings. We believe these revisions lead to a clearer articulation of the study’s motivation and a more precise interpretation of its theoretical significance.

Introduction L46-93

Humans possess a remarkable ability to rapidly infer generalizable rules from only a few examples and to apply these rules to novel inputs, a capacity known as few-shot learning (Nelli et al., 2023; Sorscher et al., 2022). This ability allows people to acquire new concepts or master new tasks with minimal experience. For example, the classic Wug Test shows that a child can correctly apply a novel label (“wug”) to new objects after exposure to only a few exemplars (Berko & Jean, 1958). Such efficiency is widely attributed to inductive bias—internal constraints that shape and restrict the learner’s hypothesis space (Lippl et al., 2024). Rather than learning solely from surface-level samples, humans actively infer and construct internal representations of underlying relational structure to make sense of incoming information.

Inductive bias is fundamentally relational: instead of treating observations as isolated events, humans tend to project causal or structural relationships onto their environment (Baxter, 2000; Lippl et al., 2024). One ubiquitous relational structure is ranking (e.g., rankings of tennis players, soccer teams, or universities), which provides an efficient means of organizing information and guiding decision-making (Dehaene et al., 2022; Iñiguez et al., 2022; Ramawat et al., 2023). Although a complete ranking formally requires either scalar values for each item or exhaustive pairwise comparisons, real-world learning typically relies on sparse samples of local pairwise relations (Jensen et al., 2021; Nelli et al., 2023; Sorscher et al., 2022; Tenenbaum et al., 2011; Xu et al., 2024). For instance, given match outcomes for only a subset of player pairs, people can infer a global ordering that includes pairs that have never directly competed.

Because ranking involves stochastic relational inference under uncertainty (Lake et al., 2015; Tenenbaum et al., 2011), we hypothesized that inductive biases are engaged during few-shot learning of local pairwise relations. Under sparse evidence and limited computational resources, relational inference is therefore not a passive accumulation of evidence but is shaped by the active construction of a global ranking structure within each individual—an account we refer to as the *constructive ranking account* (Fig 1a, right). According to this account, individuals actively construct a unified and idiosyncratic global ranking from few-shot experience, which serves as an inductive bias to constrain learning of local pairs. As a result, even when exposed to identical local pairwise information, individuals tend to form stable and self-consistent, yet idiosyncratic, global rankings that deviate from the ground truth. This account contrasts sharply with the classical *independent-value account* (Fig 1a, left), previously used to explain transitive inference (Ciranka et al., 2022; Jensen et al., 2015). Under this account, each item is represented and updated independently as a point on a latent value axis, with no constraints imposed by a global ranking structure.

A classical framework for studying relational learning is transitive inference (Acuna & Sanes, 2002; Bryant & Trabasso, 1971; Piaget, 1923; Vasconcelos, 2008), in which relationships between non-adjacent items (e.g., A < C) are inferred from relationships between adjacent items (e.g., A < B, B < C). A related phenomenon, list linking (Nelli et al., 2023; Treichler & Van Tilburg, 1996; Wei et al., 2014), occurs when two independently learned sequences are rapidly integrated into a single ordered list after learning a single bridging relation. Numerous computational and neural network models have been proposed to account for such relational learning (Ciranka et al., 2022; Delius & Siemann, 1998; Jensen et al., 2015; Mannella & Pezzulo, 2024; Wynne, 1995; Kay et al., 2024; Miconi & Kay, 2025; Mitchell, 2007; Nelli et al., 2023). Here, we adapted the transitive inference paradigm within a few-shot learning framework to directly examine the role of inductive bias in relational inference.

Discussion L331-357

Our findings largely replicate classical phenomena in transitive inference at the group level, including the canonical serial position and symbolic distance effects—hallmark signatures of relational learning observed across species and paradigms (Bryant & Trabasso, 1971; Vasconcelos, 2008). Notably, these effects emerged despite a fundamental departure from traditional designs: whereas classical paradigms rely on extensive training with adjacent pairs and continuous feedback (Ciranka et al., 2022; Jensen et al., 2015), our participants received only brief few-shot exposure to largely non-adjacent pairs, followed by testing without feedback. Importantly, these group-level effects could also be well captured by classical computational models based on incremental value updating mechanisms (Ciranka et al., 2022; Jensen et al., 2015; Vasconcelos, 2008; Wynne, 1995).

Beyond replicating canonical effects, our results substantially extend previous work on transitive inference by revealing stable, self-consistent, yet idiosyncratic ranking patterns at the individual level. These individual-level structures cannot be readily explained by classical value-updating models, highlighting a dissociation between group-level regularities and individual-level inference. This divergence suggests that incremental value updating alone is insufficient to capture the full nature of relational inference, particularly under few-shot conditions. Rather than passively accumulating evidence and updating items independently, the observed global self-consistency across local pairs points to the active imposition of a relational schema during learning. This *constructive ranking account* aligns with theoretical frameworks emphasizing structural priors (Kemp & Tenenbaum, 2008; Tenenbaum et al., 2011) and geometry-based representations of ranking (Di Antonio et al., 2024; Mannella & Pezzulo, 2024; Nelli et al., 2023; Wen et al., 2025). From this perspective, relational learning does not merely tune local values, but involves the construction of a global relational structure that constrains and supports internal consistency within each individual.

4. Was the serial position effect and symbolic distance effect (Figure 1 e + f) calculated including the learned pairs or only the unlearned pairs?

**Reply:** In the original submission, the serial position effect and symbolic distance effect shown in original Figure 1e–f were calculated by pooling all 28 pairs, including both learned and inferred pairs. In response to the reviewer’s concern, we have now conducted these analyses separately for learned and inferred pairs.

As shown below, both the serial position effect and the symbolic distance effect remain robust when calculated within each subset. The primary difference between learned and inferred pairs lies in the overall accuracy level, with learned pairs showing higher accuracy. Importantly, the characteristic patterns—enhanced accuracy for extreme serial positions and for pairs with larger rank distances—are preserved in both learned and inferred pairs.

These new analyses have been added (Figure R9, added as panels of S3 Fig).

Figure R9. Position and symbolic distance effects in learned and unlearned pairs.

5.The MEG RSA-results are reported only after averaging or subtracting. It would be important to judge the raw betas before and after the learning phase. Please add time series (like 4 g + i) with pre- and post-learning betas to Figure 4.

**Reply:** Thank you for the suggestion. We have now separately plotted the raw beta coefficient time courses for Model RDM 1 (real-world size rank) and Model RDM 2 (subject-specific rank) during the pre-learning and post-learning phases (Figure R6).

Notably, the β₂ coefficients for Model RDM 2 during the post-learning phase alone reach nominal statistical significance, although they do not survive correction for multiple comparisons (cluster-based permutation test, *p* =0.13). As clarified in our response to Reviewer 2, the beta weights during the pre-learning phase serve as a critical within-subject baseline. Subtracting pre-learning from post-learning therefore allows us to isolate neural reorganization specifically attributable to learning, rather than correlations already present prior to learning.

We have added rationales for using the pre-learning phase as a baseline (Results L289-297), and added the raw beta coefficients for both pre-learning and post-learning phases as a new panel in revised Figure 5 (b) for clarity.

Figure R6. Raw beta coefficients for both pre-learning and post-learning phases.

Results L289-297

This procedure yielded participant-specific β coefficients that quantified the strength of neural encoding for each relational structure. For real-world size, we averaged the corresponding β coefficients across the pre- and post-learning phases, reflecting its persistent representation as innate knowledge. Critically, for subjective ranking, we computed the difference between post- and pre-learning coefficients, β(post) – β(pre), to isolate neural reorganization specifically attributable to few-shot learning. This controls for pre-existing alignment between intrinsic neural geometry and the to-be-learned structure, ensuring that the observed effects reflect learning-induced changes rather than baseline correlations.

6. In the MEG experiment participants were excluded for poor learning performance. This needs to be explained in more detail. How did the results change, if these participants are included?

**Reply:** Thank you for the methodological question. We acknowledge that the original manuscript did not provide sufficient detail regarding participant exclusion criteria.

Participants with below-chance performance (accuracy < 50% across all 28 pairwise judgments) were excluded from analysis. This exclusion criterion was preregistered for the behavioral experiment (<https://osf.io/dpxq4>) and was also applied to the MEG experiment (“Participants demonstrating chance-level performance (accuracy < 0.5 across all 28 pairwise judgments) will be excluded from analyses.”). In the MEG study, 3 out of 30 participants met this criterion and were therefore excluded from further analyses.

Following the reviewer’s suggestion, we additionally reanalyzed the MEG data including all 30 participants. Under this analysis, the key effect—neural representations aligning with subject-specific rankings in the 685–775 ms time window—no longer reached statistical significance (Figure R10; *p* = 0.089, cluster-based permutation test; Peak at 720 ms, *t*(26)=3.74, *p*_unc_ < 0.001). This result is consistent with the interpretation that including participants who failed to learn the task introduces substantial noise, thereby reducing sensitivity to learning-related neural reorganization present in successful learners.

We have revised the Methods section to explicitly clarify the exclusion criterion and its preregistration (L713-715).

Figure R10. Grand-averaged time-resolved RSA results including all participants.

Results L248-249

Finally, we conducted magnetoencephalography (MEG) recordings using an Elekta NEUOROMAG system in a new cohort of participants (N = 30; three excluded due to **below-chance** learning performance)

Methods L825-826

Participants of below-chance average accuracy (<0.5) were excluded from analysis as pre-registered.

Typos:

l85: „consistent global orders that deviates from",

l385 "summary, out study highlights"

Corrected

if the Q-learning model can be reasonably fit using the learning trials and the test trials to jointly model the learning rate and the choice parameter (temperature) even though they were not experienced together. The authors cite Ciranka et al. (2022) (https://www.nature.com/articles/s41562-021-01263-w) to back up their method. However, as far as I understand that paper, the model was fit on learning data that included a choice: "All experiments involved learning the latent relations between the eight stimuli (A > B > C > D > E > F > G > H) through pairwise choice feedback, where the latent value structure was pseudo-randomly assigned to the pictures for each participant. On each trial, a pair of pictures was presented, and the observers were asked to choose the higher-valued stimulus (two-alternative choice with time-out)."

I have not found a paper, where the approach of the authors was used. My main concern is that the softmax-Choice used to prevent the model to choose the largest value option does not make sense for the test data. The reason is that the softmax-Choice and its temperature in a Rescorla-Wagner-Type-Model is enabling the exploration-exploitation-trade-off. This means that the model can still learn, even if the initial trials are biased because there is a chance that the lower value option is chosen and the system receives new feedback. However, in the case where only a test is performed and no feedback is given, the model does not have an incentive to explore, but should always choose the highest value option. It makes no sense here to use a computational model that assumes a softmax decision and, hence, it seems unsurprising the authors find the poor fit between the human data and the model data.

**Reply:** Thank you for the methodological concerns.

First, although no previous study has employed exactly the same experimental design and modeling approach, each component of our framework builds on well-established methodological foundations. Ciranka et al. (2022), for example, similarly used delta-rule learning to model value updating, albeit in a paradigm that included choices with binary feedback, whereas our learning phase presented continuous distance information without requiring choice. Despite these differences, both paradigms permit the computation of prediction errors and incremental value updating based on the same underlying principle.

Importantly, substantial empirical evidence demonstrates that value learning can occur through passive observation. For instance, McClure et al. (2003, Neuron) and Burke et al. (2010, PNAS) showed that prediction error signals and learning can occur in vicarious learning contexts. More broadly, reinforcement learning frameworks have been successfully applied to settings without immediate or action-contingent feedback in studies of structural and relational learning (e.g. Momennejad et al., 2020, Current Opinion in Behavioral Sciences; Stachenfeld et al., 2017, Nat Neurosci; Kahn et al., 2025, PLOS Computational Biology). Our paradigm represents a natural extension of this literature: participants directly observe relational distances rather than inferring them from binary feedback, while the delta-rule learning mechanism remains theoretically and empirically grounded.

Most crucially, the model captures the general behavioral patterns observed in our data, including overall accuracy levels and canonical transitive inference phenomena such as the symbolic distance effect and the serial position effect. This indicates that the basic value-updating framework provides a valid computational account of how participants process relational information in our task. The model’s limitation arises specifically in accounting for the systematic construction of individually coherent global rankings, which is precisely the novel dimension revealed by our study.

Second, the reviewer questioned whether the *softmax* choice policy is appropriate in a testing phase without feedback, and whether the mismatch between model and data might be driven by this choice rule. To directly address this concern, we implemented a deterministic variant of the model by setting the inverse temperature parameter to an extremely large value (10⁸), thereby eliminating choice stochasticity and forcing the model to always select the higher-valued option. If the softmax policy were inappropriately introducing noise that obscures model performance, this deterministic version should improve model–data correspondence.

Instead, as shown below, we observed the opposite pattern: the deterministic model achieved near-perfect performance, with most simulated participants making correct judgments on all pairs. This outcome is expected, as Q-learning successfully recovers the full ranking structure from the eight learned pairs. Critically, even under optimal, noise-free decision rules, the model still fails to reproduce our central empirical findings—namely, systematic inter-individual differences and globally coherent error patterns. This demonstrates that the softmax decision rule is not responsible for the model’s inability to capture the observed data.

More generally, we note that the exploration-exploitation framework is not applicable to our testing context. Exploration-exploitation tradeoffs arise specifically in multi-armed bandit scenarios where choosing one option prevents observation of alternatives, creating genuine information asymmetry that softmax helps resolve. Transitive inference differs fundamentally: participants receive identical relational info rmation regardless of their response, as value updating proceeds from observed pairwise relationships rather than choice-contingent feedback. This mirrors the approach in Jensen et al. (2015) and Ciranka et al. (2022), where Q-values update based on presented stimulus pairs independent of participant choices. In such contexts, softmax serves not to balance exploration and exploitation but to model inherent decision noise—a standard application in decision neuroscience. For instance, Kable and Glimcher (2007, Nature Neuroscience) employed softmax for intertemporal choices where no learning opportunity exists, while logistic regression—mathematically equivalent to softmax—represents the default framework for binary choice modeling precisely because it captures decision variability independent of learning dynamics.

We have added the deterministic model analysis to the Results (L228-231; Figure R11 added as S11 Fig) and clarified the empirical validity and theoretical foundations of our modeling approach.

Figure R11. Q-learning simulation with deterministic choices.

Results L228-232

All the models predominantly produced correct global rankings accompanied by self-inconsistent local errors, rather than the incorrect global rankings yet self-consistent local errors observed in the majority of human participants (Fig 3b, right; S6 Fig; see S11 Fig for deterministic simulation of Q-learning)

Discussion L332-342

Our findings largely replicate classical phenomena in transitive inference at the group level, including the canonical serial position and symbolic distance effects—hallmark signatures of relational learning observed across species and paradigms (Bryant & Trabasso, 1971; Vasconcelos, 2008). Notably, these effects emerged despite a fundamental departure from traditional designs: whereas classical paradigms rely on extensive training with adjacent pairs and continuous feedback (Ciranka et al., 2022; Jensen et al., 2015), our participants received only brief few-shot exposure to largely non-adjacent pairs, followed by testing without feedback. Importantly, these group-level effects could also be well captured by classical computational models based on incremental value updating mechanisms

Discussion L345-352

These individual-level structures cannot be readily explained by classical value-updating models, highlighting a dissociation between group-level regularities and individual-level inference. This divergence suggests that incremental value updating alone is insufficient to capture the full nature of relational inference, particularly under few-shot conditions. Rather than passively accumulating evidence and updating items independently, the observed global self-consistency across local pairs points to the active imposition of a relational schema during learning.

Discussion L380-387

Rather, our results point to a mechanism largely absent from current frameworks – the human brain actively imposes a global relational structure to maintain internal coherence, even at the cost of making systematic errors on specific local comparisons. Instead of passively accumulating independent pairwise evidence, participants construct unified ranking schemas guided by inductive biases—internal constraints that shape the hypothesis space and enforce transitivity. This constructive process underscores the active, generative nature of human relational learning under sparse samples and cognitive resource limitations.

**Journal Requirements:**

1) Please amend your detailed Financial Disclosure statement. This is published with the article. It must therefore be completed in full sentences and contain the exact wording you wish to be published.

1.State the initials, alongside each funding source, of each author to receive each grant. Done.

2. State what role the funders took in the study. If the funders had no role in your study, please state: “The funders had no role in study design, data collection and analysis, decision t”. Done.

2) Please upload the main figures as separate figure files in .tif or .eps format. For more information about figure files please see our guidelines:

https://journals.plos.org/plosbiology/s/figures

https://journals.plos.org/plosbiology/s/figures#loc-file-requirements

Done.

3) We have noticed that you have uploaded Supporting Information files, but you have not included a list of legends. Please add a full list of legends for your Supporting Information files after the references list.

Done.

4) Please refer to the following article for PLOS Biology's best-practice recommendations for data presentation: https://journals.plos.org/plosbiology/article?id=10.1371/journal.pbio.1002128.

Guidelines for statistical reporting, if relevant, may be found here: https://journals.plos.org/plosbiology/s/best-practices-in-research-reporting#loc-statistical-reporting.

5) Some material included in your submission may be copyrighted. According to PLOS’s copyright policy, authors who use figures or other material (e.g., graphics, clipart, maps) from another author or copyright holder must demonstrate or obtain permission to publish this material under the Creative Commons Attribution 4.0 International (CC BY 4.0) License used by PLOS journals. Please closely review the details of PLOS’s copyright requirements here: PLOS Licenses and Copyright. If you need to request permissions from a copyright holder, you may use PLOS's Copyright Content Permission form.

Please respond directly to this email and provide any known details concerning your material's license terms and permissions required for reuse, even if you have not yet obtained copyright permissions or are unsure of your material's copyright compatibility. Once you have responded and addressed all other outstanding technical requirements, you may resubmit your manuscript within Editorial Manager.

Potential Copyright Issues:

-1a,1b,1c, 2a,2d,2f,4a,4b,4f,4g,4i,s1b,s2d, and s2f: Please confirm whether you drew the images / clip-art within the figure panels by hand. If you did not draw the images, please provide (a) a link to the source of the images or icons and their license / terms of use; or (b) written permission from the copyright holder to publish the images or icons under our CC BY 4.0 license. Alternatively, you may replace the images with open source alternatives. See these open source resources you may use to replace images / clip-art:

- https://commons.wikimedia.org

- <https://openclipart.org/>

Those images are acquired from <http://www.bcbl.eu/databases/multipic>, and are open-access and free from copyright restrictions for non-commercial purposes as stated (Duñabeitia et al, 2018).

If you have any questions or require assistance, please don't hesitate to get in touch via return email. To help us minimize inbox volumes, we kindly ask that you not reply to this email simply to confirm you have resubmitted your revisions.
