# Supplementary material for: Human brains construct individualized global rankings from identical few-shot learning input
Source: PLoS Biol. 2026 Apr 13;24(4):e3003756. doi: 10.1371/journal.pbio.3003756 (PMC13108901; doi:10.1371/journal.pbio.3003756)
Supplement: Attachment — Submitted filename: LiuWangLuo.2026.ResponsetoReviews2.v4.docx [file pbio.3003756.s015.docx]

Responses to reviewers

## Reviewer #1

The authors have addressed all of my original concerns. I have no further comments.

## Reviewer #2

All my comments have been addressed.

**Reply:** We thank the two reviewers for their time and for confirming that all concerns have been addressed. We are grateful for the constructive feedback, which has substantially improved the manuscript.

## Reviewer #3

### Q1

I would like to thank the authors for their clear explanation of the process. I did understand now much better the underlying ideas. However, I remain doubtful. This is because in absence of choice at learning, how should the fitted values functions differ between different participants? This would be crucial for idiosyncratic errors. Maybe I am missing something, but it seems logical that the models do not have idiosyncrasies if they all had the exact same learning experience. I mean, if they do, I would be eager to see these data. Humans on the other hand will have fluctuating attention and thus all differ between each in the information they have access to leading to idiosyncratic errors (see my comment 3).

**Reply:** We thank the reviewer for this important point. We fully agree that the observed idiosyncratic global rankings arising from identical local pair experience cannot be explained by incremental Q-learning. Indeed, this limitation is precisely what motivated the computational modeling analysis in our manuscript.

Importantly, we would like to clarify that, despite its inability to account for idiosyncratic ranking structures, the Q-learning model is still able to capture inter-individual differences in averaged accuracy (shown below), which may arise from variations in attention or encoding noise, even under identical learning conditions.

We agree with the reviewer that attentional fluctuations and trial-to-trial encoding variability are potentially natural sources of individual differences in behavior, and we do not dispute this point. Indeed, as stated in our Introduction and Abstract, it is precisely under conditions of sparse evidence and limited computational resources — which naturally encompass attentional variability and imperfect encoding — that we propose the brain engages in constructive ranking. In this sense, noise does not undermine our account, but rather characterizes the condition under which such constructive processes become necessary.

Critically, however, Q-learning—by virtue of its independent item-updating framework—cannot account for the emergence of idiosyncratic yet internally consistent global rankings (92.8% of participants exhibited zero circular triads). If behavior were primarily driven by encoding noise or forgetting, one would expect less constrained and more inconsistent responses, leading to *more* circular triads rather than fewer. The near absence of circular triads is therefore difficult to reconcile with a noise-based account alone.

We have revised the Discussion accordingly to clarify this point, copied below:

L185-188:

“Notably, despite its inability to account for idiosyncratic ranking structures, the Q-learning model is able to capture inter-individual differences in averaged accuracy, even under identical learning conditions”

L383-392:

“One might argue that idiosyncratic rankings simply reflect attentional fluctuations, imperfect encoding, or random forgetting across individuals—factors that naturally arise under limited computational resources, particularly in few-shot learning. However, the observed pattern of self-consistent errors cannot be explained by merely introducing stochasticity or idiosyncratic attentional fluctuations into traditional independent-value frameworks. Critically, the remarkably low rate of circular triad errors in local pairwise comparisons indicates that errors are not independent noisy distortions, but are instead systematically constrained by each individual’s subjectively constructed global ranking. This global structure cannot be captured by stochastic attentional parameters acting independently on each local pair.”

### Q2

Thank you for clarifying.

**Reply:** Thanks！

### Q3

I think this is crucial. The novelty of the finding is based on the authors' claiming it not being modelled by the computational models, but these models do not include things like attention. Maybe a fix would be to extend the model to include attention effects, e.g., by fitting the Q-learning and allowing missing trials (possibly even as a fit-able parameter). Possibly this would lead to models being idiosyncratic, too? In any case a positive model would strongly convince me that the claim the authors are making is true.

**Reply:** We appreciate the concern that is closely related to Q1. Specifically, the reviewer doubt that the failure of classical model for idiosyncratic observations might be due to the lack of individualized attentional parameter in the model.

Regarding the suggestion to extend the Q-learning model with missing trials (i.e., stochastic forgetting), we appreciate the intuition behind this proposal. Meanwhile, we would respectfully argue that this could not account for our major findings either. If participants randomly failed to encode certain pairs during learning, this could plausibly lead to inter-individual variability ranking performance *but theoretically could not account for the observed self-consistency pattern*, the crucial component of constructive ranking after few-shot learning. *In other words, the self-consistency findings implicate a global structure constraint underlying individual item updating,* *which could not be captured by stochastic attentional parameter exerted on each pair learning.*

Moreover, if participants inconsistently encoded certain pairs due to fluctuating attention, their responses on those pairs would be less constrained but more variable — which would be expected to increase the rate of circular triads, not reduce it. In other words, random forgetting would introduce stochastic inconsistency, as shown in our model fitting, whereas our empirical data displays structured, self-consistency within each individual's idiosyncratic ranking.

From an implementation perspective, a truly random forgetting mechanism — one that does not assume which trials are more or less likely to be encoded — would also be difficult to constrain with a single interpretable parameter, making it challenging to test in a principled way within our trial-by-trial fitting framework.

We have added more clarification to address the point, copied below:

L383-392:

“One might argue that idiosyncratic rankings simply reflect attentional fluctuations, imperfect encoding, or random forgetting across individuals—factors that naturally arise under limited computational resources, particularly in few-shot learning. However, the observed pattern of self-consistent errors cannot be explained by merely introducing stochasticity or idiosyncratic attentional fluctuations into traditional independent-value frameworks. Critically, the remarkably low rate of circular triad errors in local pairwise comparisons indicates that errors are not independent noisy distortions, but are instead systematically constrained by each individual’s subjectively constructed global ranking. This global structure cannot be captured by stochastic attentional parameters acting independently on each local pair.”

### Q4

Wow. Great to see ϑ It might be worth calculating stats on the slope of the symbolic distance effect. It seems larger in the non-learned pairs, which seems logical, but nonetheless is an interesting finding!

**Reply:** We thank the reviewer for this suggestion. We apologize for not explicitly noting this in our previous response letter. The slope comparison was in fact already implemented: as shown in the updated S3 Fig (panel b), we report the slope coefficients separately for learned and non-learned pairs, along with corresponding statistical tests. As the reviewer anticipated, the symbolic distance effect is steeper for non-learned pairs (slope = 0.05, *t*(76) = 14.141, *p* < 0.001) than for learned pairs (slope = 0.02, *t*(76) = 4.556, *p* < 0.001).

### Q5

Thank you for providing pre- and post-data. Judging from these curves, it does not seem that there is a distinct difference between the two curves and significance emerges due to the subtraction procedure. I would urge the authors to clearly point this out in the limitations section and to inform readers that additional research is required. Out of curiosity, does the non-significant cluster permutation test for the post subjective ranking yield a cluster at the same time point? This would strengthen the authors' claim!

**Reply:** Thank you. We have incorporated this limitation into the Results (copied below). Regarding the timing issue, the post-learning beta coefficients for subjective ranking reveal a nominally significant cluster within a similar time window, although this effect does not survive cluster-based permutation correction (*p* = 0.13).

L315-319: “Notably, the subjective ranking effect during the post-learning phase, when analyzed without pre-learning baseline correction, shows a nominally significant peak within a similar temporal window (Fig. 5b; peak at 890 ms, t(26) = 2.80, uncorrected p = 0.005), but does not survive cluster-based permutation correction (p = 0.13), indicating reduced sensitivity when geometric confounds are not controlled.”

### Q6

Add the info to the ms that the effects are non-significant, if all participants are included. As the authors point out, this is in line with expectations.

**Reply:** We thank the reviewer for this suggestion. Following the reviewer's advice, we have now explicitly noted in the Results, copied below:

L319:324: “Moreover, the subjective ranking effect does not reach significance when the three excluded participants with below-chance behavioral performance are included (*p* = 0.089, cluster-based permutation test). This pattern is consistent with the expectation that unsuccessful learners introduce noise into the data, thereby reducing sensitivity to learning-related effects.”
